# Efficacy of severe fever with thrombocytopenia syndrome vaccines in animal models: a systematic review and meta-analysis

**DOI:** 10.3389/fmicb.2026.1978600

**Published:** 2026-10-13

**Authors:** Eric Sibomana, Eurade Ntakiyisumba, Fabrice Hirwa, Maryum Tanveer, Noutlady Inthavong, Jaime C. Cabarles, John Hwa Lee, Gayeon Won

**Affiliations:** 1 Bio-Safety Research Institute and College of Veterinary Medicine, Jeonbuk National University, Iksan, Republic of Korea; 2 College of Agriculture, Resources and Environmental Science, Central Philippine University, Iloilo, Philippines

**Keywords:** animal model, meta-analysis, SFTSV, systematic review, tick-borne disease, vaccine efficacy, zoonotic

## Abstract

Severe fever with thrombocytopenia syndrome (SFTS) is an emerging tick-borne zoonosis that threatens both animal and human health across East Asia, yet no licensed vaccine is currently available. We therefore conducted a PRISMA-guided systematic review and meta-analysis of preclinical murine and ferret studies to evaluate severe fever with thrombocytopenia syndrome virus vaccine efficacy. Seven electronic databases were searched for studies published between 2009 and 2025. Random-effects meta-analyses evaluated mortality, viremia, and post-challenge weight loss. Quantitative viral burden was further evaluated using standardized mean differences for mean and peak viremia, whereas body-weight trajectories were assessed using generalized linear mixed models. Twenty-three studies met the inclusion criteria. Vaccination markedly reduced mortality (RR = 0.10, 95% CI 0.05–0.21), post-challenge weight loss (RR = 0.21, 95% CI 0.12–0.37), and detectable viremia (RR = 0.45, 95% CI 0.27–0.73), although the effect estimate was attenuated in the trim-and-fill sensitivity analysis (RR = 0.74, 95% CI: 0.36–1.51). Furthermore, significantly suppressing both mean and peak viremia under homologous and heterologous challenge conditions. Longitudinal analyses further demonstrated improved preservation of body weight, particularly following mRNA and vector vaccination. These findings provide the strongest quantitative evidence supporting the development of broadly protective SFTSV vaccines for both human and veterinary use within a One Health framework.

## 1 Introduction

Severe fever with thrombocytopenia syndrome (SFTS) is an emerging zoonotic tick-borne disease caused by severe fever with thrombocytopenia syndrome virus (SFTSV), a bandavirus first identified in human patients in China in 2009 and has subsequently been reported extensively in South Korea and Japan (Liu et al., 2014; Yu et al., 2011). Since its discovery, it has become recognized as a major public health threat with important animal health and One Health implications, with human cases concentrated predominantly in rural and hilly areas where competent tick vectors are present. In humans, SFTSV infection typically presents after a 7–14-day incubation period with acute fever, fatigue, headache, myalgia, and gastrointestinal symptoms. Laboratory findings commonly include thrombocytopenia, leukopenia, and elevated liver enzymes, lactate dehydrogenase, and creatine kinase; severe cases may develop hemorrhagic and neurological manifestations, coagulopathy, and multiorgan failure. Diagnosis is primarily based on detection of SFTSV RNA in blood by reverse-transcription PCR or real-time RT-PCR during the acute phase, while serological detection of SFTSV-specific IgM/IgG can provide supportive or retrospective confirmation.

Companion animals, particularly cats, can develop severe and often fatal disease, with a case-fatality rate of 62.5% reported among 24 SFTS-confirmed cats in Japan (Matsuu et al., 2019; Ishijima et al., 2022). Although dogs are frequently infected subclinically, severe disease and localized outbreaks can occur, and molecular evidence confirms both dog-to-human bites and dog-to-cat transmission (Mekata et al., 2022; Kim et al., 2025). In addition to vector-borne transmission, direct animal-to-human and nosocomial transmission from sick cats has repeatedly infected veterinary personnel, with genomic evidence supporting transmission pathways (Yamanaka et al., 2020). Risk of infection is also further associated with exposure to tick-infested environments, particularly among farmers. Collectively, these observations underscore the urgent need for animal-focused vaccination strategies to protect both animal and human populations (Oshima et al., 2022; Han et al., 2022).

Beyond companion animals, livestock and other vertebrate hosts play major ecological roles in maintaining SFTS transmission. High seroprevalence has been reported in goats, sheep, cattle, and dogs, supporting their function as amplifying hosts that maintain virus-tick cycles near farms and villages (Chen et al., 2019; Xu et al., 2024). Modeling studies further suggest that non-human hosts are central drivers of viral maintenance in nature, highlighting the importance of reducing viremia in animals as a strategy to limit spillover risk (Cheng et al., 2025). From a veterinary public health standpoint, even subclinical infections in livestock matter because they sustain infected tick populations that then threaten animals and people working around them (Cheng et al., 2025). The primary vector, *Haemaphysalis longicornis*, is native to eastern Asia and has been documented in China, Japan, South Korea, Russia, India, Australia, New Zealand, the USA, New Caledonia, and Vanuatu. It serves as an important vector of SFTSV and can facilitate virus transmission during blood feeding, with evidence of transovarial and transstadial transmission (Zhuang et al., 2018; Zhao et al., 2020; Randolph et al., 1996).

Despite the substantial One Health burden posed by SFTSV, a licensed veterinary vaccine is not yet available. Preclinical vaccine development has therefore relied heavily on murine models, generating a diverse range of candidate platforms with promising efficacy. However, given the distinctive biology of SFTSV, including its reliance on Gn/Gc glycoproteins for cell entry, ability to suppress type I interferon signaling, and marked age-dependent pathogenesis, appropriate models are essential for evaluating vaccine-induced protection (Sun et al., 2023). Wild-type mice typically develop only mild disease due to limited viral replication, restricting their utility for evaluating clinical protection (Chen et al., 2012). Consequently, most studies employ interferon-deficient systems, such as IFNAR-knockout or IFNAR-blockade models, which recapitulate key features of severe disease, including high viremia, cytopenia, organ involvement, and rapid lethality (Liu et al., 2014; Matsuno et al., 2017). Beyond mice, aged ferrets represent the most clinically relevant model, reproducing age-dependent severity and systemic disease patterns observed in humans and companion animals (Park et al., 2019). Aged ferrets develop high fever, profound cytopenia, multiorgan viremia, and high mortality following SFTSV challenge, whereas young ferrets remain asymptomatic, closely mirroring real-world susceptibility patterns (Yu et al., 2019; Jin et al., 2012).

Several strategies for developing SFTSV vaccines have been suggested, including DNA vaccines encoding the Gn and Gc glycoproteins, which have repeatedly induced neutralizing antibodies and conferred survival benefits in lethal-challenge models (Kwak et al., 2019). Viral-vectored vaccines, including recombinant vesicular stomatitis virus (rVSV), vaccinia-based constructs, and adenoviral vectors, have shown strong immunogenicity, rapid onset of protection, and robust reductions in viremia (Kim et al., 2023c; Tian et al., 2021; Dong et al., 2019). Protein vaccines using purified Gn, Gc, or Gn–Gc fusion proteins formulated with aluminum hydroxide, CpG, or saponin adjuvants have demonstrated dose-dependent protection and cross-strain neutralization (Kim et al., 2024; Kim et al., 2023a). Virus-like particles (VLPs) expressing SFTSV glycoproteins elicit potent humoral and cellular responses, while mRNA vaccines targeting the Gn/Gc antigens are among the most promising candidates, with several studies reporting complete survival and durable immunity in mice following lethal challenge (Lu et al., 2024; Kim et al., 2023d). Ferret studies, though fewer, similarly demonstrate that Gn/Gc-directed protein and vectored vaccines can prevent clinical disease and suppress viral replication, supporting cross-species relevance of these platforms (Yu et al., 2019; Kim et al., 2023a). Collectively, these experimental findings illustrate a mature and heterogeneous murine and ferret evidence base for SFTSV vaccine development.

In contrast to the extensive murine literature, vaccine development in veterinary target species remains in its early stages. Only a small number of studies have evaluated prototype vaccines in dogs, including an inactivated whole-virus vaccine that successfully blocked viremia and prevented histopathological lesions after challenge (Park et al., 2024). In cats, the species with the highest case fatality rate, experimental vaccine studies are virtually absent, and no candidates have progressed to clinical evaluation (Hwang et al., 2017). This imbalance between robust murine evidence and limited data in target species represents a major barrier to translational progress. To bridge this gap, there is a clear need for a structured appraisal of the murine literature to identify which antigens, platforms, and immunization strategies demonstrate the strongest and most consistent protection, and which candidates warrant prioritization for translation into veterinary species.

Against this background, the present systematic review and meta-analysis aimed to (i) generate pooled estimates of vaccine efficacy against mortality, viremia, and clinical outcomes in murine and ferret models; (ii) characterize sources of heterogeneity in vaccine efficacy reported across vaccination trials; and (iii) evaluate vaccine platforms to derive evidence-based design principles for canine and feline vaccine development. Addressing these gaps is critical given the growing disease burden in animals, the reservoir role of livestock, and the expanding geographic range of SFTSV driven by ecological and climatic changes.

## 2 Methods

### 2.1 Study design

A systematic review and meta-analysis was conducted in accordance with the 2020 guidelines of the Preferred Reporting Items for Systematic Reviews and Meta-Analyses (PRISMA) (Page et al., 2020). The objective was to synthesize and quantitatively evaluate the preclinical evidence on the efficacy of SFTSV vaccine candidates in mouse and ferret models. The research question was formulated using the PICO (population, intervention, comparator, and outcome) framework. The “population” of interest consisted of mice or ferrets. The “intervention” included vaccination with any SFTSV vaccine candidate, irrespective of platform, including but not limited to mRNA, protein, DNA, inactivated, vector, and live-attenuated vaccines. “Comparators” consisted of unvaccinated controls (animals receiving nothing, a placebo, or a sham vaccine). Moreover, the “outcomes” of interest were clinical disease outcomes, including mortality, viremia, and clinical disease indicators, particularly body weight loss.

### 2.2 Literature search strategy

A comprehensive literature search was performed across multiple electronic databases, including PubMed, Scopus, Web of Science, along with several Korean databases, including Korean Studies Information Service System (KISS), Research Information Sharing Service (RISS), ScienceOn, and ScienceDirect, covering publications from 2009 (corresponding to the first SFTS report in China) (Yu et al., 2011) to 2025 with no language restrictions. In addition, supplementary searches were conducted in Google Scholar to identify potentially relevant gray literature (theses, institutional/government reports). The search strategy incorporated Boolean operators and controlled vocabulary where applicable, combining terms related to SFTSV, vaccination, animal models, and protective efficacy. The core search string was as follows: (severe fever with thrombocytopenia syndrome OR severe fever with thrombocytopenia OR SFTS OR SFTSV OR SFTV OR Dabie bandavirus OR bandavirus OR Huaiyangshan banyangvirus OR banyangvirus) AND (vaccine OR vaccination OR immunization OR immun* OR DNA OR mRNA OR viral vector OR viral-vector OR inactivated OR killed OR attenuat* OR live-attenuated OR live attenuated OR protein OR virus-like particle* OR VLP OR nanoparticle) AND (mouse OR mice OR murine OR ferret*) AND (efficacy OR effect* OR protection OR survival). Reference lists of all eligible studies and relevant review articles were manually screened using a backward snowballing strategy to identify additional potentially eligible publications. All retrieved records were subsequently imported into EndNote 20 (Clarivate Analytics) for reference management, organization, and duplicate removal before the screening process.

### 2.3 Eligibility and exclusion criteria

Studies were considered eligible if they evaluated the efficacy of any SFTSV vaccine candidate in murine or ferret models, with both vaccinated and control groups subjected to experimental challenge with virulent SFTSV. Eligible studies were required to be published between 2009 and 2025. In addition, the eligible study must have reported at least one prespecified efficacy outcome with extractable group-level data, including mortality, viremia, or body weight loss. Preprints and gray literature were also included if they met all eligibility criteria and provided sufficient methodological detail and quantitative data for extraction. Contrarily, studies were excluded if they were not conducted in mice or ferrets, involved non-vaccine interventions, lacked a concurrent control or comparator group for efficacy assessment, or described observational or natural-exposure infections without experimental challenge. Reviews, editorials, commentaries, and protocols without primary data were also excluded. Additional exclusion criteria included studies with insufficient numerical data to compute effect sizes, experiments involving co-interventions administered at or after viral challenge that could confound vaccine efficacy estimates, and duplicate reports that lacked unique data. All identified records underwent title and abstract screening, followed by full-text review to determine eligibility.

### 2.4 Data extraction

The data extraction was performed by two independent reviewers to minimize bias, with disagreements resolved through discussion and consensus. A standardized data extraction form was developed in Microsoft Excel and piloted before use. Extracted data included study identifiers (first author, year of publication, and country); animal model features (species, strain, age, sex, and immune status); vaccine characteristics (platform, antigen composition, formulation and adjuvant, dose, route of administration, and immunization schedule); study design (number of animals per experimental group, time of challenge, challenge strain, challenge dose, route, and time to follow-up); and post-challenge clinical outcomes (mortality, viremia, and body weight loss). From each group, the number of events for dichotomous outcomes and the mean and standard deviation (SD) for continuous outcomes were extracted. In some studies where outcomes were reported graphically, WebPlotDigitizer-Version 4.8 (Ankit Rohatgi, Pacifica, CA, USA) was applied to extract data from graphs (Drevon et al., 2017). When a single study evaluated multiple vaccination trials, each trial was treated as a separate comparison only when interventions differed in vaccine platform or antigen; trials involving minor variations within the same platform or formulation were combined into a single intervention group. For the overall pooled analyses of mortality, viremia, and weight loss, multiple eligible vaccine groups sharing a common control were combined into a single study-level comparison. For prespecified subgroup analyses by vaccine type, antigen, dose, or route of administration, separate intervention groups were retained, and shared control groups were proportionally allocated across comparisons to avoid double-counting while preserving the total number of control animals and events (Higgins and Green, 2024).

### 2.5 Quality assessment and risk of bias

The external and internal validity of included studies were assessed using the ARRIVE (Animal Research: Reporting of *in vivo* Experiments) 2.0 guidelines checklist (Percie du Sert et al., 2020). All 18 ARRIVE 2.0 items were assessed. Each item was rated as low, unclear, or high risk of bias based on the information reported in the respective study. The assessment was conducted by three independent reviewers, with discrepancies resolved through consensus.

### 2.6 Statistical analysis of data

The efficacy of SFTSV vaccines was evaluated using three key outcomes: mortality, viremia, and post-challenge weight loss. Mortality was analyzed as a dichotomous outcome, with pooled effect estimates expressed as risk ratios (RRs) and corresponding 95% confidence intervals (CIs), comparing the risk of death between vaccinated and control groups. Viremia was analyzed as both a binary and a continuous outcome. Detectable viremia was defined as the presence or absence of detectable virus at the end of the experimental period according to the assay-specific detection criteria reported in each study. Viremia levels were quantitatively compared between vaccinated and control groups using standardized mean differences (SMDs) with Hedges’ *g* correction for small-sample bias (Borenstein et al., 2010). Two complementary measures of viremia levels were evaluated: mean viremia, calculated as the average viral titer across all reported time points, and peak viremia, defined as the highest reported mean viral titer. Viremia levels were further analyzed separately for homologous and heterologous challenge strains. To evaluate the impact of vaccination on disease severity, post-challenge body weight loss was analyzed as a dichotomous outcome, defined as the presence or absence of weight loss at the last reported follow-up time point. In addition, longitudinal body weight trajectories were analyzed using a generalized linear mixed model (GLMM), with vaccine platform, challenge type, and days post-challenge included as fixed effects. Estimated marginal means (EMMs) and their corresponding 95% confidence intervals were calculated from the fitted model to describe body weight trajectories over time according to vaccine platform and challenge type. Higher estimated body weight values were interpreted as reduced disease-associated weight loss and improved clinical protection following vaccination. All statistical analyses were conducted in R (version 4.1.2) using RStudio (version 1.4) with the “meta,” “metafor,” and “dmetar” packages (Balduzzi et al., 2019; Harrer et al., 2021; Team, 2014; Viechtbauer, 2010). For dichotomous outcomes, vaccine efficacy (VE) was calculated using the formula (1-RR)*100, representing the percentage reduction in the risk of the outcome attributable to vaccination (De Oliveira et al., 2022). Corresponding 95% CIs for VE were derived using the substitution method (Daly, 1998). The robustness of the pooled estimates was evaluated through sensitivity analyses, including leave-one-out analyses and exclusion of studies identified as statistical outliers. Between-study heterogeneity was assessed using the Cochran Q test and quantified by the *τ*^2^ and *I*^2^ statistics (Higgins et al., 2003; IntHout et al., 2016). Where sufficient data were available, 95% prediction intervals were calculated to reflect the expected dispersion of true effects across experimental settings (Daly, 1998).

Despite expected between-study variability arising from differences in animal models, vaccine platforms, antigens, challenge conditions, and experimental protocols, studies were considered sufficiently conceptually comparable to permit quantitative synthesis of prespecified outcomes. Random-effects models were used to account for anticipated variation in true effects across experimental settings. To investigate potential sources of heterogeneity, subgroup analyses were conducted according to vaccine platform (mRNA, protein, DNA, vector, live-attenuated, and inactivated vaccines) and animal model (IFNAR-deficient, IFNAR-impaired, aged ferrets, wild-type immunized, and wild-type humanized). For each vaccine type with sufficient studies, additional exploratory subgroup analyses examined antigen composition, vaccination dosage frequency, and route of administration. Potential publication bias was evaluated using funnel plots and Egger’s regression test (Sabarimurugan et al., 2019). When evidence of publication bias was detected, the Duval and Tweedie trim-and-fill method was applied to estimate an unbiased pooled effect by imputing potentially missing studies (Sabarimurugan et al., 2019; Duval and Tweedie, 2000).

## 3 Results

### 3.1 Search results

The systematic literature search identified 638 records from database searches, with an additional six records identified through other sources, including reference list screening. After removal of duplicates, 577 records were screened by title and abstract, of which 480 were excluded. Ninety-seven full-text articles were assessed for eligibility, and 74 were excluded due to irrelevant content, lack of outcomes of interest, absence of accessible full text, or non-murine/ferret study design. Ultimately, 23 studies met the inclusion criteria and were included in both the qualitative synthesis and quantitative meta-analysis as summarized in the PRISMA flow diagram (Figure 1A).

**Figure 1:**
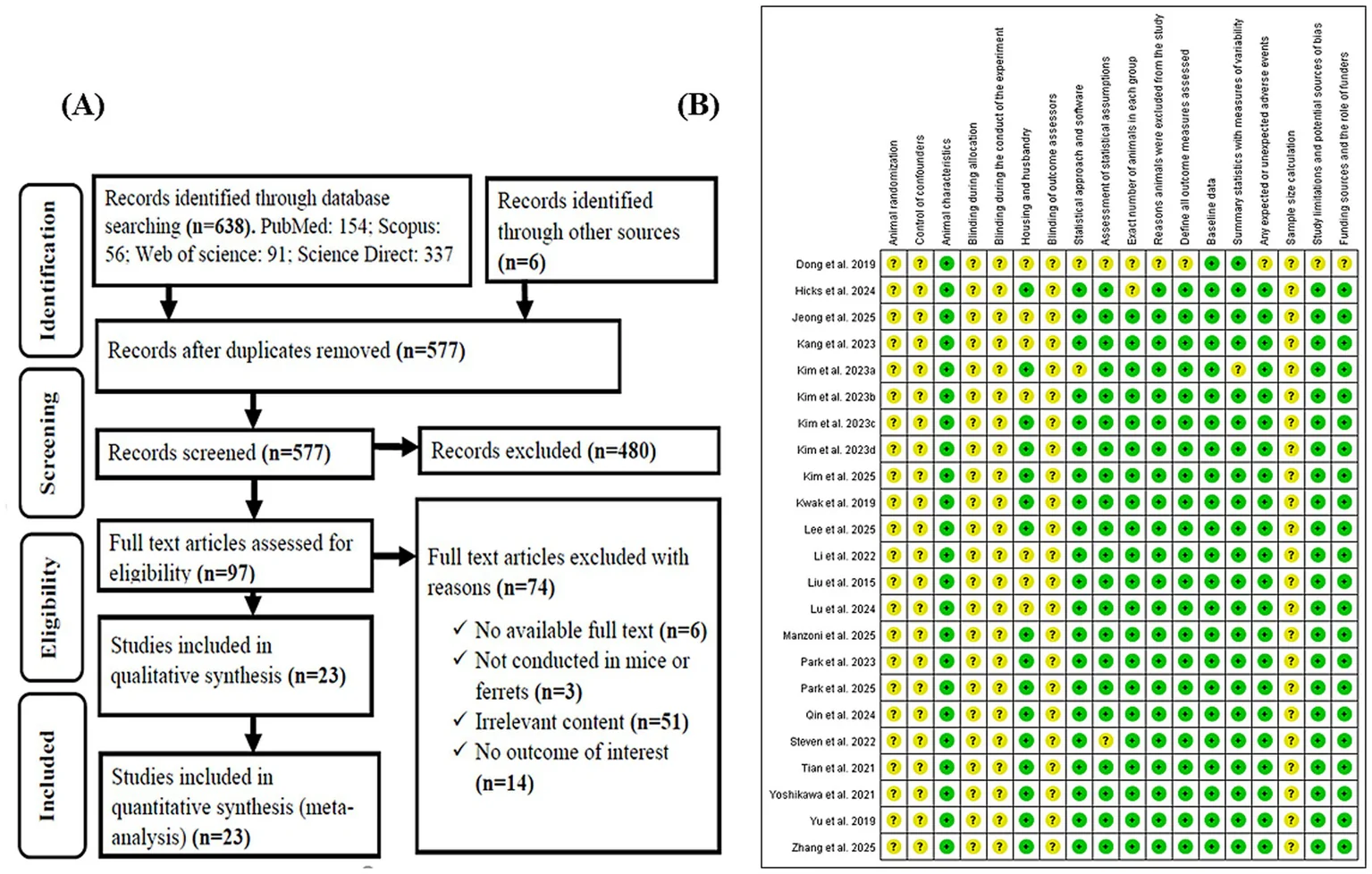
PRISMA flow diagram of the selection of studies for use in a systematic review and meta-analysis of the efficacy of SFTSV Vaccines in Mice and Ferrets **(A)**. Risk of bias assessment of eligible studies using the ARRIVE checklist **(B)**.

### 3.2 Study characteristics

After a comprehensive evaluation of the identified records, 23 studies met the inclusion criteria. The primary characteristics of these studies are summarized in Table 1. Most studies were conducted in East Asia, with South Korea (*n* = 11 studies) and China (*n* = 7 studies) contributing the largest proportion, followed by the United States (*n* = 3), the United Kingdom (*n* = 1), and Japan (*n* = 1). A diverse range of vaccine platforms was evaluated across the included studies. Vector vaccines were the most frequently investigated (*n* = 8) (Kim et al., 2023c; Tian et al., 2021; Dong et al., 2019; Qian et al., 2024; Yoshikawa et al., 2021; Hicks et al., 2024; Park et al., 2025; Park et al., 2023), followed by mRNA vaccines (*n* = 6) (Lu et al., 2024; Kim et al., 2023d; Jeong et al., 2025; Kim et al., 2023b; Manzoni et al., 2025; Zhang et al., 2025), protein vaccine (*n* = 3) (Kim et al., 2024; Kim et al., 2023a; Liu et al., 2015), inactivated vaccines (*n* = 2) (Li et al., 2022; Lee et al., 2025), live-attenuated vaccines (*n* = 2) (Yu et al., 2019; Bryden et al., 2022), and DNA vaccine (*n* = 1) (Kwak et al., 2019). One study evaluated a combination of DNA and protein vaccines (Kang et al., 2020). Vaccine antigens predominantly targeted the SFTSV surface glycoproteins Gn and/or Gc. Administration routes varied, with intramuscular and subcutaneous routes being the most common. Immunization frequency ranged from single-dose regimens to multi-dose prime–boost strategies, with most studies employing two doses before viral challenge. Challenge experiments differed with respect to SFTSV strain, challenge dose, route of infection, and the interval between the last vaccination and viral challenge, reflecting heterogeneity in experimental designs.

**Table 1:** Characteristics of studies included in the systematic review and meta-analysis of the efficacy of the SFTSV vaccine in mice and ferrets.

Author/year	Country	Animal model	Vaccine type	Vaccine antigen	Vaccine dose	Vaccination route
Jeong et al. (2025)	South Korea	IFNAR-deficient^1^	mRNA	Gn + Gc	2-doses	Intramuscular
Kim et al. (2023a)	South Korea	Aged ferrets^2^	Protein-based	Gn	3-doses	Intramuscular
Kim et al. (2023b)	USA	IFNAR-deficient	mRNA	Gn	2-doses	Intramuscular
Kim et al. (2023c)	South Korea	IFNAR-impaired^3^	Vector-based	Gn	2-doses	Intramuscular
Kim et al. (2023d)	USA	IFNAR-impaired	mRNA	Gn	2-doses	Intramuscular
Lu et al. (2024)	China	IFNAR-deficient	mRNA	Gn + Gc	1-dose	Intramuscular
Manzoni et al. (2025)	USA	IFNAR-impaired	mRNA	Gn + Gc	1-dose, 2-doses	Intradermal
Zhang et al. (2025)	China	IFNAR-deficient	mRNA	Gc	2-doses	Intramuscular
Kim et al. (2024)	South Korea	IFNAR-deficient	Protein-based	NP, Gn, Gc, NP + Gn, NP + Gc, Multi-antigens	3-doses	Subcutaneous
Liu et al. (2015)	China	WT-immunocompetent^4^	Protein-based	NSs	2-doses	Subcutaneous
Kang et al. (2020)	South Korea	IFNAR-deficient	DNA-based; Protein-based	Gn, Gc, Multi-antigens	3-doses	Intramuscular
Kwak et al. (2019)	South Korea	Aged ferrets	DNA-based	Gn + Gc, Multi-antigens	3-doses	Intradermal
Bryden et al. (2022)	UK	IFNAR-deficient	Live-attenuated	Whole virus	1-dose	Intraperitoneal
Yu et al. (2019)	South Korea	Aged ferrets	Live-attenuated	Whole virus	1-dose	Intramuscular
Li et al. (2022)	China	WT-immunocompetent	Inactivated	Whole virus	3-doses	Intramuscular
Lee et al. (2025)	South Korea	Aged ferrets	Inactivated	Whole virus	2-doses	Intramuscular
Dong et al. (2019)	China	IFNAR-deficient	Vector-based	Gn + Gc	1-dose	Intraperitoneal, Intravenous, Subcutaneous, Intranasal
Hicks et al. (2024)	USA	IFNAR-deficient	Vector-based	Gn + Gc	1-dose	Intraperitoneal
Park et al. (2023)	South Korea	WT-humanized^5^	Vector-based	NSs, Multi-antigens	2-doses	Intramuscular
Park et al. (2025)	South Korea	WT-humanized	Vector-based	Gn + Gc	1-dose	Intramuscular
Qian et al. (2024)	China	IFNAR-impaired	Vector-based	Gn, Gc, Gn + Gc	1-dose	Intramuscular
Tian et al. (2021)	China	WT-immunocompetent	Vector-based	Gn	1-dose	Intramuscular
Yoshikawa et al. (2021)	Japan	IFNAR-deficient	Vector-based	NP, Gn + Gc, Multi-antigens	2-doses	Subcutaneous

IFNAR, interferon-α/β receptor; WT, Wild Type; NR, Not Reported. ^1^IFNAR knockout mice lacking type I interferon receptor signaling. ^2^Ferrets aged ≥3 years. ^3^Mice with impaired type I interferon signaling. ^4^Wild-type immunocompetent mice with intact immune function. ^5^Humanized wild-type mice expressing human immune components.

In all experiments, animal models were standardized based on mechanisms of susceptibility to SFTSV (Table 2). Accordingly, vaccine efficacy was evaluated predominantly in genetic IFNAR knockout mouse models (*n* = 10), followed by immunocompetent wild-type mouse models, with or without receptor humanization (*n* = 5), antibody-mediated IFNAR blockade models (*n* = 4), and ferrets aged ≥3 years (*n* = 4) (Table 2). In addition, the follow-up characteristics were broadly comparable, with observation periods ranging from a minimum of 4 days to a maximum of 30 days (Supplementary Table S1).

**Table 2:** Summary of characteristics of the animals used in the preclinical studies included in the review.

Author	Species	Strain	Animal model	Gender	Age
Jeong et al. (2025)	Mice	C57BL/6	IFNAR-deficient^1^	Female	8 weeks
Lu et al. (2024)	Mice	A129	IFNAR-deficient	NR	6–10 weeks
Manzoni et al. (2025)	Mice	C57BL/6	IFNAR-impaired^2^	Male, Female	4 weeks
Zhang et al. (2025)	Mice	C57BL/6	IFNAR-deficient	Female	6–8 weeks
Kim et al. (2023a)	Ferrets	NR	Aged ferrets^3^	NR	>4 years
Kim et al. (2023b)	Mice	A129	IFNAR-deficient	Female	6–8 weeks
Kim et al. (2023c)	Mice	C57BL/6	IFNAR-impaired	Female	6 weeks
Kim et al. (2023d)	Mice	C57BL/6	IFNAR-impaired	Female	6 weeks
Kim et al. (2024)	Mice	C57BL/6	IFNAR-deficient	Female	6–8 weeks
Liu et al. (2015)	Mice	C57BL/6 J	WT-immunocompetent^4^	Female	3-4 weeks
Kang et al. (2020)	Mice	C57BL/6	IFNAR-deficient	Female	6–8 weeks
Kwak et al. (2019)	Ferrets	NR	Aged ferrets	NR	>4 years
Bryden et al. (2022)	Mice	A129	IFNAR-deficient	Male, Female	8–12 weeks
Yu et al. (2019)	Ferrets	NR	Aged ferrets	NR	>4 years
Li et al. (2022)	Mice	C57BL/6	WT-immunocompetent	Female	NR
Lee et al. (2025)	Ferrets	NR	Aged ferrets	Male	3 years
Dong et al. (2019)	Mice	C57BL/6	IFNAR-deficient	Male, Female	6–8 weeks/ 8–9 months
Hicks et al. (2024)	Mice	C57BL/6	IFNAR-deficient	Male, Female	8 weeks
Park et al. (2023)	Mice	C57BL/6	WT-humanized^5^	Female	5 weeks
Park et al. (2025)	Mice	C57BL/6	WT-humanized	Female	5 weeks
Qian et al. (2024)	Mice	BALB/c	IFNAR-impaired	Female	6–8 weeks
Tian et al. (2021)	Mice	C57BL/6	WT-immunocompetent	Female	6–8 weeks
Yoshikawa et al. (2021)	Mice	C57BL/6	IFNAR-deficient	Male, Female	6–9 weeks

IFNAR, interferon-α/β receptor; WT, Wild Type; NR, Not Reported. ^1^IFNAR knockout mice lacking type I interferon receptor signaling. ^2^Mice with impaired type I interferon signaling. ^3^Ferrets aged ≥3 years. ^4^Wild-type immunocompetent mice with intact immune function. ^5^Humanized wild-type mice expressing human immune components.

### 3.3 Quality assessment

The risk-of-bias assessment across the 23 included studies was evaluated using all 18 items of the ARRIVE 2.0 guidelines. No study was judged to be at low risk of bias across all assessed domains, primarily because several methodological domains were insufficiently reported (Figure 1B). No domain was classified as high risk across the included studies. Reporting of animal characteristics, including species, strain, age, and sex, was generally complete, and all studies were considered to be at a low risk of bias. In contrast, none of the studies explicitly discussed the animal randomization procedures, blinding during allocation, and blinding during conduct of the experiment, resulting in all studies being rated as having unclear risk in these domains. Most studies adequately reported housing and husbandry information, while reporting of blinding of outcome assessors was predominantly unclear. Statistical methods and software, assessment of statistical assumptions, the exact number of animals in each group, definition of outcome measures, baseline data, and summary statistics with measures of variability were generally well reported and predominantly rated as low risk. Reasons for animal exclusions and reporting of unexpected adverse events were also adequately addressed in most studies. The included studies did not report sample-size determination and were therefore consistently classified as unclear risk. Study limitations and potential sources of bias were generally reported, although some studies provided insufficient information. Funding sources and the role of funders were also generally reported and did not indicate a high risk of bias.

### 3.4 Meta-analysis results

#### 3.4.1 Mortality outcome

In the overall meta-analysis, vaccination was associated with a substantial reduction in mortality following SFTSV challenge (RR = 0.107; 95% CI: 0.054–0.213), corresponding to an overall vaccine efficacy of 89.3% (95% CI: 78.7–94.6). However, there was considerable between-study heterogeneity (*I*^2^ = 73.8%; *τ*^2^ = 0.9373; *p* < 0.0001; Figure 2A). A leave-one-out sensitivity analysis identified three studies (Kim et al., 2024; Qian et al., 2024; Kang et al., 2020) as potential outliers contributing disproportionately to between-study variability (Figure 2B). To investigate their influence on the pooled effect size, the analysis was repeated after excluding these studies. When these studies were excluded, the pooled effect estimate became even more pronounced, shifting from a VE of 89.3 to 92.4% (RR = 0.076; 95% CI: 0.046–0.126). The between-study heterogeneity also markedly reduced from *I*^2^ = 73.8% (*p* < 0.01) to *I*^2^ = 0% (*p* = 0.54). These findings suggest that the observed heterogeneity was primarily driven by these three studies with extreme effects, while the direction and magnitude of the vaccine-associated survival benefit were consistent across the remaining studies.

**Figure 2:**
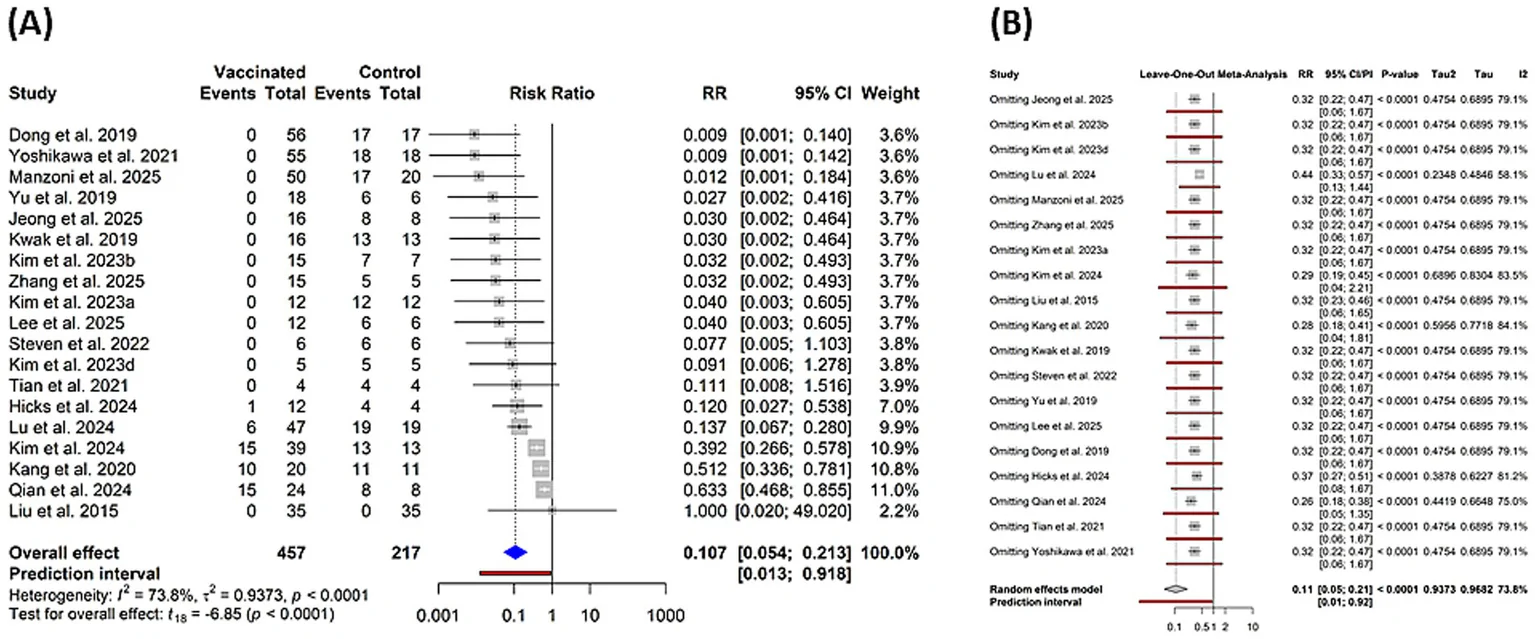
Forest Plot of the efficacy of SFTSV vaccines based on mortality outcome **(A)**. Leave-one-out analysis for mortality outcome **(B)**. The pooled effect estimate is presented as the RR with its corresponding 95% confidence interval, calculated using a random-effects model.

Additionally, subgroup analyses compared effect sizes stratified by vaccine platform and animal model. A statistically significant difference in effect size was observed among vaccine platforms (*χ*^2^ = 11.88, *p* = 0.0364), with favorable protective estimates observed for mRNA and vector vaccines (Figure 3A). Similarly, subgroup analyses revealed significant differences in effect estimates across animal models (*χ*^2^ = 10.14, *p* = 0.017). The greatest protective effects were observed in IFNAR-deficient mice and aged ferrets, whereas effect estimates for antibody-mediated IFNAR blockade mice and immunocompetent wild-type mice were less precise because of the limited number of contributing studies (Figure 3B).

**Figure 3:**
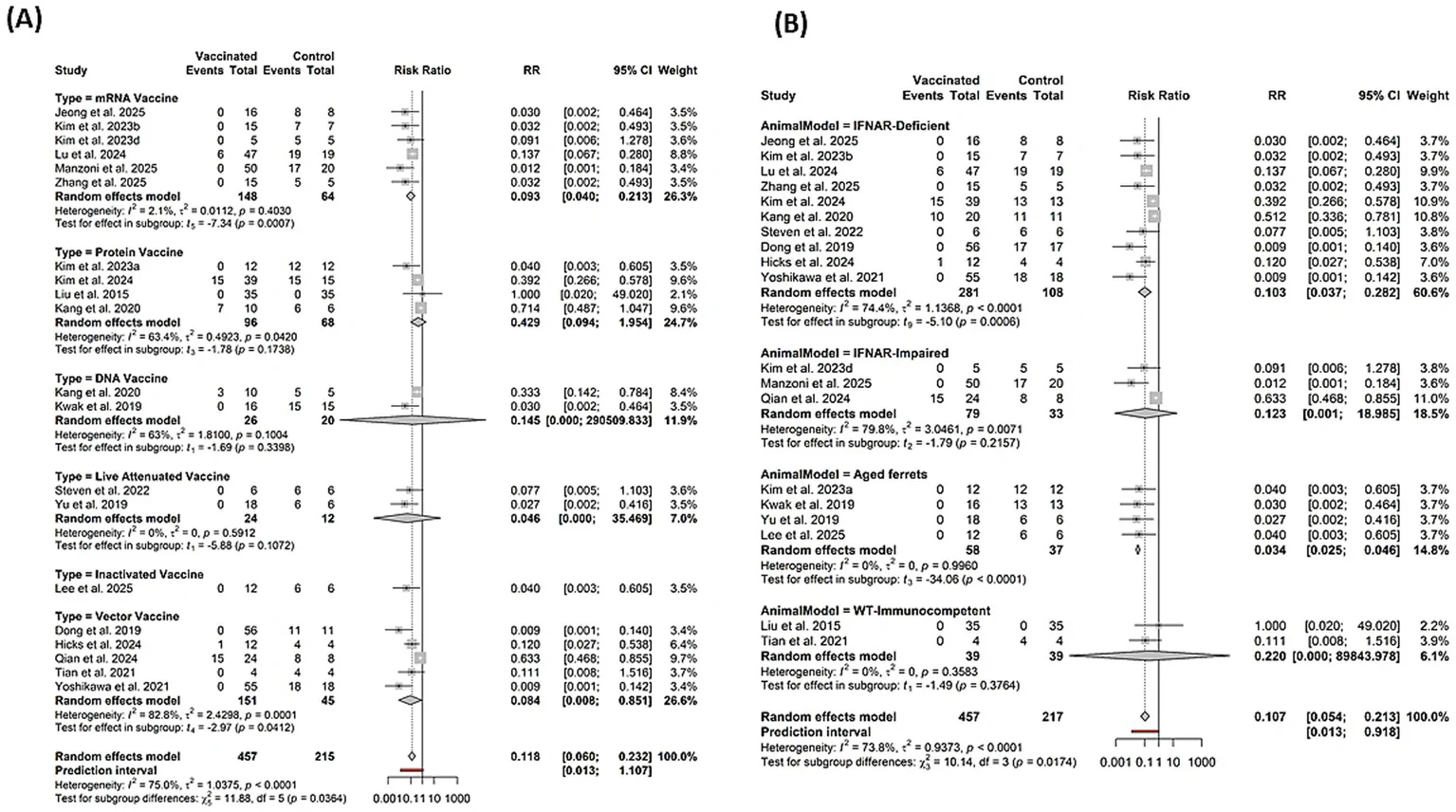
Forest plot of subgroup analysis based on vaccine type **(A)**. Forest plot of subgroup analysis based on the animal model **(B)**. The pooled effect estimate is presented as the RR with its corresponding 95% confidence interval, calculated using a random-effects model.

Given the favorable protective estimates observed for mRNA and vector vaccines, further exploratory subgroup analyses assessed the effects of dosage frequency, route of administration, and antigen composition on mortality outcomes (Table 3). Among mRNA vaccines, two-dose regimens showed more consistent mortality reduction than single-dose regimens, although subgroup differences were not statistically significant (*χ*^2^ = 0.84; *p* = 0.358). Intramuscular administration was associated with a significant reduction in mortality, whereas the intradermal estimate was based on a single study and therefore remains highly uncertain; subgroup differences were not statistically significant (*χ*^2^ = 2.37; *p* = 0.123). Vaccines containing Gn antigens, alone or combined with Gc, generally favored improved survival; however, confidence intervals were wide, and subgroup differences were not significant (*χ*^2^ = 0.18; *p* = 0.913).

**Table 3:** Exploratory subgroup analysis of mRNA and Vector vaccines by vaccine antigen, dosage frequency, and vaccination route for the mortality outcome.

Variables	Vaccine type	Subgroups	K^a^	Effect size estimates		Heterogeneity	
				RR (95% CI)	*p*-value	*I* ^2^	*p*-value
Vaccine antigen	mRNA	Gn + Gc	3	0.067 (0.001–5.345)	0.1172	73.9	0.0217
		Gn	2	0.055 (0.000–39.624)	0.1125	0	0.592
		Gc	1	0.032 (0.002–0.493)	-	-	-
	Vector-based	Gn + Gc	4	0.101 (0.004–2.519)	0.108	86.9	<0.0001
		Gn	2	0.082 (0.001–4.631)	0.0803	0	0.7396
		Gc	1	1.000 (0.788–1.268)	-	-	-
		NP	1	0.026 (0.002–0.396)	-	-	-
		Multi-antigens	1	0.027 (0.002–0.416)	-	-	-
Vaccine dosage	mRNA	1 dose	2	0.153 (0.0–30153742.7)	0.43	78.3	0.0317
		2 doses	6	0.038 (0.025–0.059)	<0.0001	0	0.987
	Vector-based	1 dose	4	0.132 (0.009–2.012)	0.0989	79.8	0.002
		2 doses	1	0.009 (0.001–0.142)	-	-	-
Vaccination route	mRNA	Intramuscular	5	0.106 (0.05–0.222)	0.001	0	0.596
		Intradermal	1	0.012 (0.001–0.184)	-	-	-
	Vector-based	Intravenous	1	0.091 (0.006–1.278)	-	-	-
		Subcutaneous	2	0.03 (0.000–70324.02)	0.202	28.9	0.235
		Intranasal	1	0.091 (0.006–1.278)	-	-	-
		Intraperitoneal	2	0.051 (0.000–67635.18)	0.2277	51.5	0.151
		Intramuscular	2	0.439 (0.000–3570.5)	0.4524	40.5	0.195

^a^Number of studies.

Among vector-based vaccines, subgroup analysis by antigen composition showed that vaccines expressing Gn + Gc or Gn alone were generally associated with reduced mortality, although substantial heterogeneity was observed and subgroup differences were significant (*χ*^2^ = 69.04, *p* < 0.0001). Two-dose regimens tended to show greater mortality reduction than single-dose regimens, but subgroup differences were not significant (*χ*^2^ = 2.66, *p* = 0.10). Subcutaneous and intraperitoneal administration were associated with lower mortality, whereas intramuscular delivery showed more variable protection, with no significant subgroup differences by route (*χ*^2^ = 5.42, *p* = 0.25).

#### 3.4.2 Viremia outcome

##### 3.4.2.1 Detectable viremia

In the overall meta-analysis, vaccination was associated with a reduction in detectable viremia at the end of the experimental period (RR = 0.451; 95% CI: 0.276–0.737; Figure 4A), corresponding to an overall vaccine efficacy of 54.9% (95% CI: 26.3–72.4). However, considerable between-study heterogeneity was observed (*I*^2^ = 74.1%; *p* < 0.0001). Sensitivity analysis (using leave-one-out method) identified two potential outlier studies (Liu et al., 2015; Kang et al., 2020; Figure 4B). When these studies were excluded from the analysis, the pooled effect estimate slightly changed from RR = 0.451 (95% CI: 0.276–0.737, VE = 54.9%) to RR = 0.385 (95% CI: 0.227–0.651, VE = 61.5%). However, the observed between-study heterogeneity remained relatively unchanged (*I*^2^ = 74.1% vs. 66.5%), indicating that the overall variability across studies persisted despite the omission of outliers.

**Figure 4:**
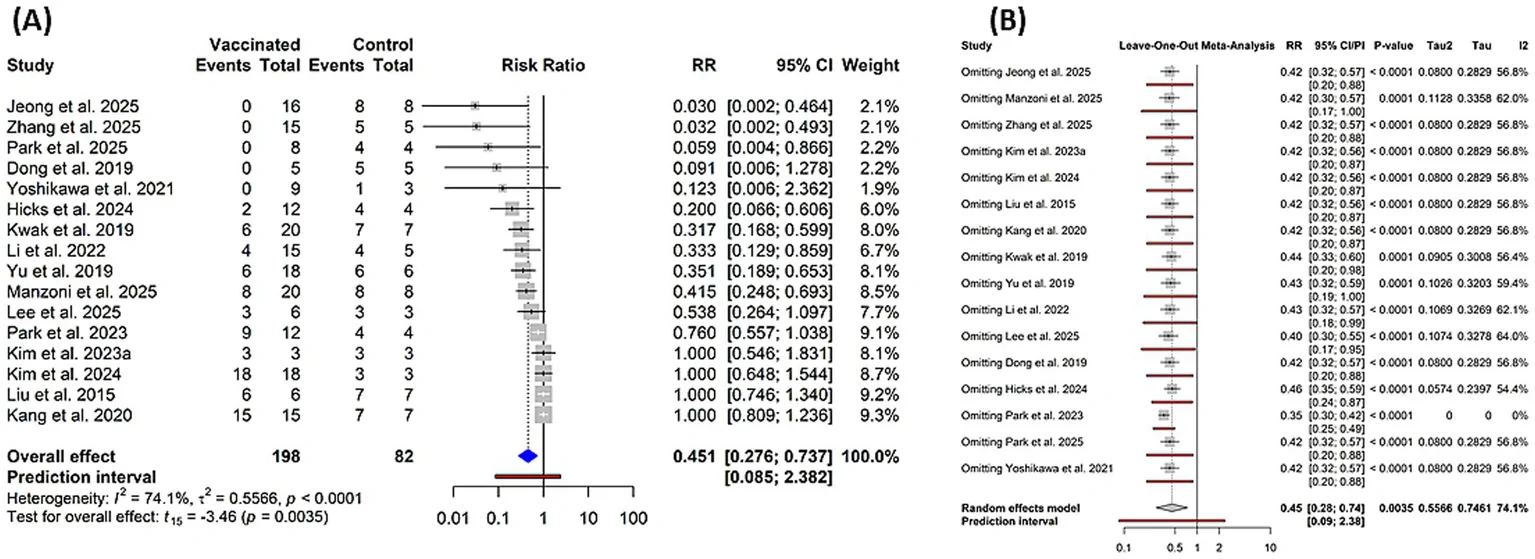
Forest Plot of the efficacy of SFTSV vaccines based on viremia outcome **(A)**. Leave-one-out analysis for viremia outcome **(B)**. The pooled effect estimate is shown as the RR with its corresponding 95% confidence interval, as calculated using a random-effects model.

To further explore sources of heterogeneity, subgroup analyses were conducted by vaccine platform and animal model. Platform-specific estimates varied, with mRNA vaccines showing favorable point estimates for detectable viremia (RR = 0.12, 95% CI 0.002–6.097), followed by viral vector vaccines (RR = 0.289, 95% CI 0.078–1.066), while protein vaccines demonstrated no protective effect (RR = 1.00, 95% CI 1.00–1.00; Figure 5A). However, differences between vaccine platforms were not statistically significant (*p* = 0.186). Similarly, vaccination appeared to reduce the risk of detectable viremia more effectively in IFNAR-deficient mice and aged ferrets than in other animal models, although subgroup differences were not statistically significant (*p* = 0.78; Figure 5B).

**Figure 5:**
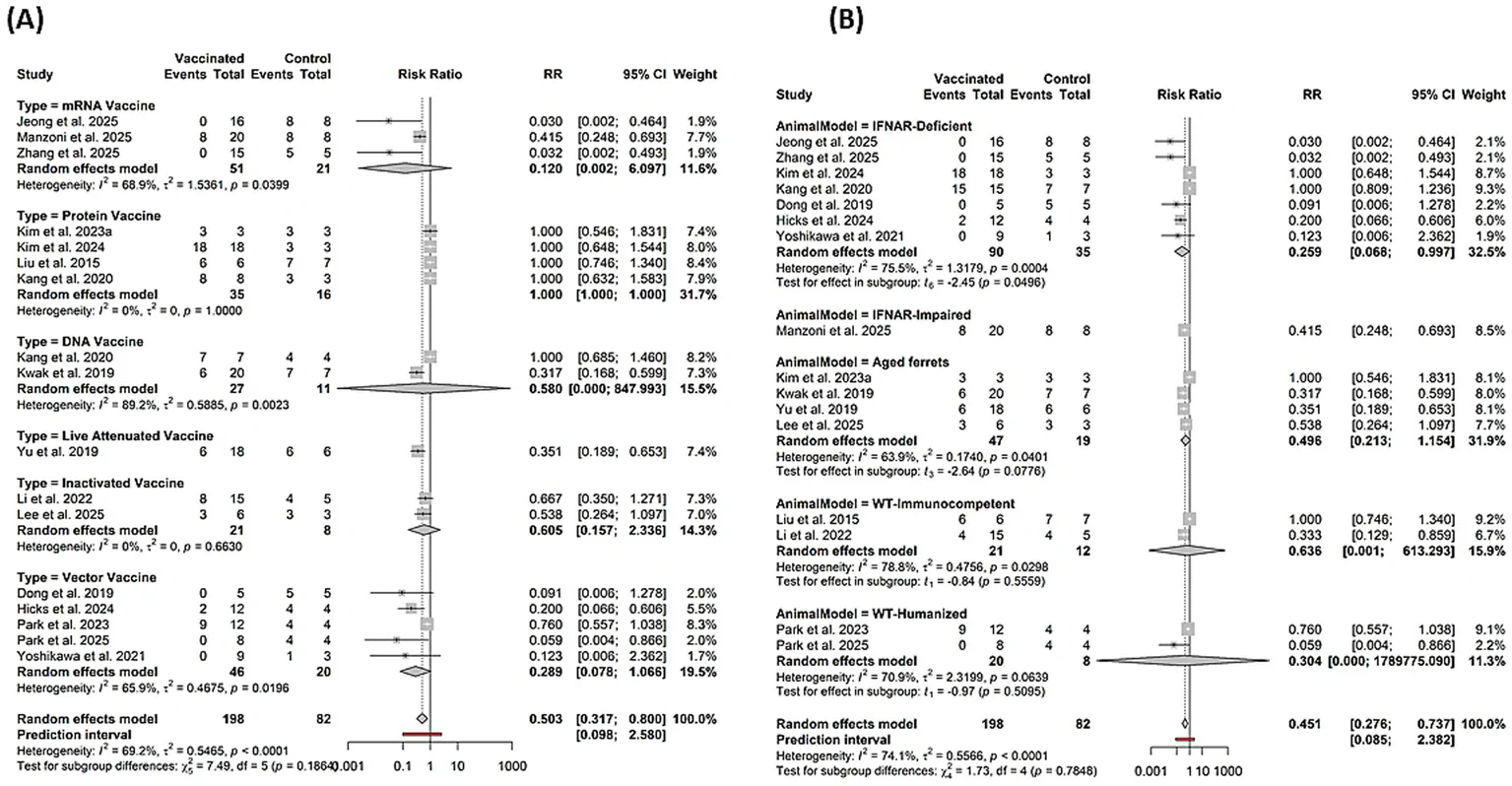
Forest Plot of subgroup analysis based on vaccine type **(A)**. Forest plot of subgroup analysis based on the animal model **(B)**. The pooled effect estimate is shown as the RR with its corresponding 95% confidence interval, calculated using a random-effects model.

Among mRNA and vector vaccines, further exploratory subgroup analyses were conducted to assess the effects of route of administration, dosage frequency, and antigen composition on viremia outcomes (Table 4). For mRNA vaccines, intramuscular administration was associated with reduced detectable viremia compared with intradermal delivery, although the latter was based on a single study. Subgroup differences by route were statistically significant (*χ*^2^ = 95.66, *p* < 0.0001). Two-dose regimens were also associated with a significant reduction in detectable viremia, with significant subgroup differences observed by dosage frequency (*χ*^2^ = 271.02, *p* < 0.0001). Antigen-based analysis suggested that Gc-containing mRNA vaccines, either alone or combined with Gn, were associated with reduced viremia, although subgroup differences were not statistically significant (*χ*^2^ = 0.73, *p* = 0.39).

**Table 4:** Exploratory subgroup analysis of mRNA and Vector vaccines by vaccine antigen, dosage frequency, and vaccination route for the viremia outcome.

Variables	Vaccine type	Subgroups	K^a^	Effect size estimates		Heterogeneity	
				RR (95% CI)	*p*-value	*I* ^2^	*p*-value
Vaccine antigen	mRNA	Gn + Gc	2	0.16 (0.0–1406978.7)	0.3833	70.7	0.064
		Gc	1	0.032 (0.002–0.493)	-	-	-
	Vector-based	Gn + Gc	4	0.167 (0.068–0.409)	0.0079	0	0.774
		NSs	1	1 (0.63–1.587)	-	-	-
		NP	1	0.333 (0.02–5.688)	-	-	-
		Multi-antigen	2	0.634 (0.152–2.654)	0.1545	0	0.651
Vaccine dosage	mRNA	1 dose	1	1 (0.693–1.444)	-	-	-
		2 doses	3	0.034 (0.024–0.049)	0.989	0	0.0006
	Vector-based	1 dose	3	0.155 (0.039–0.611)	0.0280	0	0.651
		2 doses	2	0.566 (0.00–2835.7)	0.5523	30.7	0.229
Vaccination route	mRNA	Intramuscular	2	0.031 (0.021–0.047)	0.974	0	0.0057
		Intradermal	1	0.415 (0.248–0.693)	-	-	-
	Vector-based	Subcutaneous	1	0.123 (0.006–2.362)	-	-	-
		Intraperitoneal	2	0.178 (0.005–6.337)	0.1028	0	0.589
		Intramuscular	2	0.304 (0.00–1,789,775)	0.5095	70.9	0.063

^a^Number of studies.

Among vector-based vaccines, constructs expressing Gn + Gc were associated with reduced detectable viremia compared to multi-antigen vaccines, with significant subgroup differences observed across antigen categories (*χ*^2^ = 25.68, *p* < 0.0001). Single-dose regimens were associated with a significant reduction in detectable viremia, whereas two-dose regimens showed greater variability, although subgroup differences were not statistically significant (*χ*^2^ = 3.06, *p* = 0.08). By route of administration, intraperitoneal delivery was associated with reduced viremia, while intramuscular and subcutaneous administration showed protective trends with greater uncertainty; subgroup differences were not statistically significant (*χ*^2^ = 0.25, *p* = 0.88).

##### 3.4.2.2 Viremia levels

Beyond the binary assessment of detectable viremia, 11 studies comprising 18 trials reported continuous measures of viremia (mean ± standard deviation), allowing evaluation of viremia levels following SFTSV challenge. Overall, vaccination significantly reduced mean viremia compared with non-vaccinated controls (SMD = −3.47; 95% CI −4.59 to −2.35), although substantial between-study heterogeneity was observed (*I*^2^ = 73.6%; Figure 6A). Notably, subgroup analysis indicated comparable reductions in mean viremia following both homologous (SMD = −3.17; 95% CI −4.47 to −1.87) and heterologous challenge (SMD = −4.3; 95% CI −6.61 to −1.99). A similar pattern was observed for peak viremia, with vaccination significantly reducing peak viral load compared with non-vaccinated controls (SMD = −6.39; 95% CI −8.40 to −4.39; Figure 6B). Unlike mean viremia, subgroup analysis showed significantly greater reductions in peak viremia following heterologous challenge (SMD = −8.76; 95% CI –12.33 to −5.19) than homologous challenge (SMD = −4.16; 95% CI -6.41 to −1.91; *p* = 0.032).

**Figure 6:**
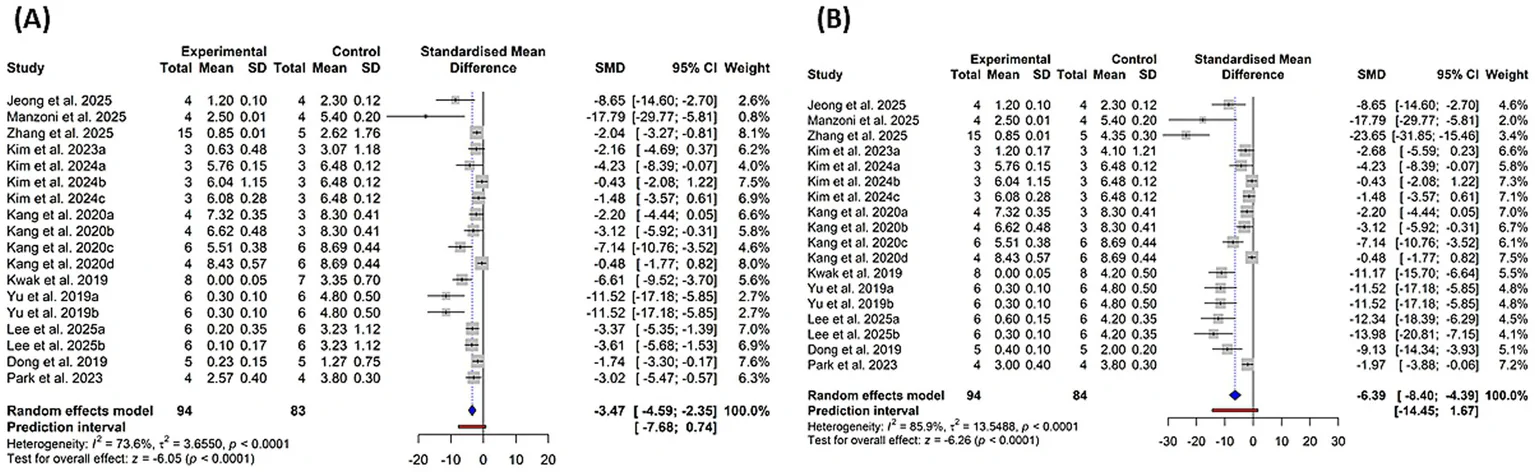
Forest plots of standardized mean differences (SMDs) in mean viremia **(A)** and peak viremia **(B)** between vaccinated and control animals following SFTSV challenge. Studies are stratified by challenge type (heterologous or homologous). Squares represent study-specific SMDs with 95% confidence intervals (CI), while diamonds indicate pooled subgroup and overall effect estimates. Negative SMD values indicate lower viremia in vaccinated animals than in controls.

#### 3.4.3 Weight loss outcome

##### 3.4.3.1 Weight loss at the end of experimental period

Across studies reporting post-challenge weight loss as a binary clinical outcome, vaccinated animals were substantially less likely to experience weight loss following SFTSV challenge (RR = 0.215; 95% CI: 0.124–0.372), corresponding to an overall vaccine efficacy of 78.5% (95% CI: 62.8–87.6). However, moderate to substantial between-study heterogeneity was observed (*I*^2^ = 68.5%; *τ*^2^ = 0.6589; *p* < 0.0001; Figure 7A). A sensitivity analysis (using the leave-one-out method) identified two studies (Kim et al., 2023c; Qian et al., 2024) that are potential outliers with extreme effect sizes (Figure 7B). Exclusion of those studies from the analysis yielded a comparable protective effect with a RR of 0.180 (95% CI: 0.103–0.313), corresponding to a VE of 82.0%. The observed between-study heterogeneity also reduced from *I*^2^ = 68.5 to 48.8%.

**Figure 7:**
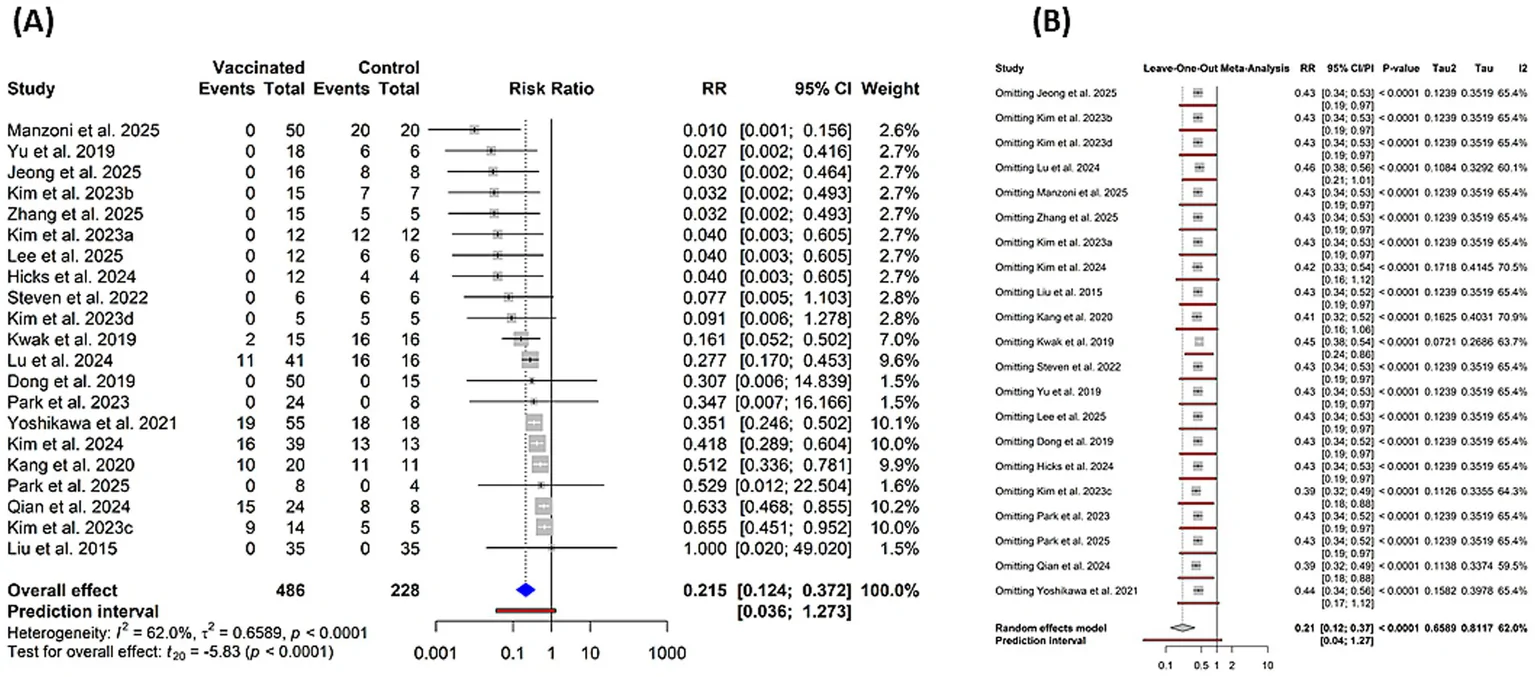
Forest Plot of the efficacy of SFTSV vaccines based on weight loss outcome **(A)**. Leave-one-out analysis for weight loss outcome **(B)**. The pooled effect estimate is presented as the RR with its corresponding 95% confidence interval, calculated using a random-effects model.

Subgroup analysis revealed that vaccine platform significantly (*p* < 0.001) influenced the magnitude of protection against weight loss following challenge, with favorable estimates observed for mRNA (RR = 0.08, 95% CI 0.02–0.318) and vector vaccines (RR = 0.502, 95% CI 0.326–0.774) (Figure 8A). Similarly, effect estimates varied across animal models, with favorable estimates observed in IFNAR-deficient, IFNAR-impaired mice, and aged ferrets (Figure 8B). However, data were limited in immunocompetent and humanized wild-type mouse models, precluding reliable pooled estimates.

**Figure 8:**
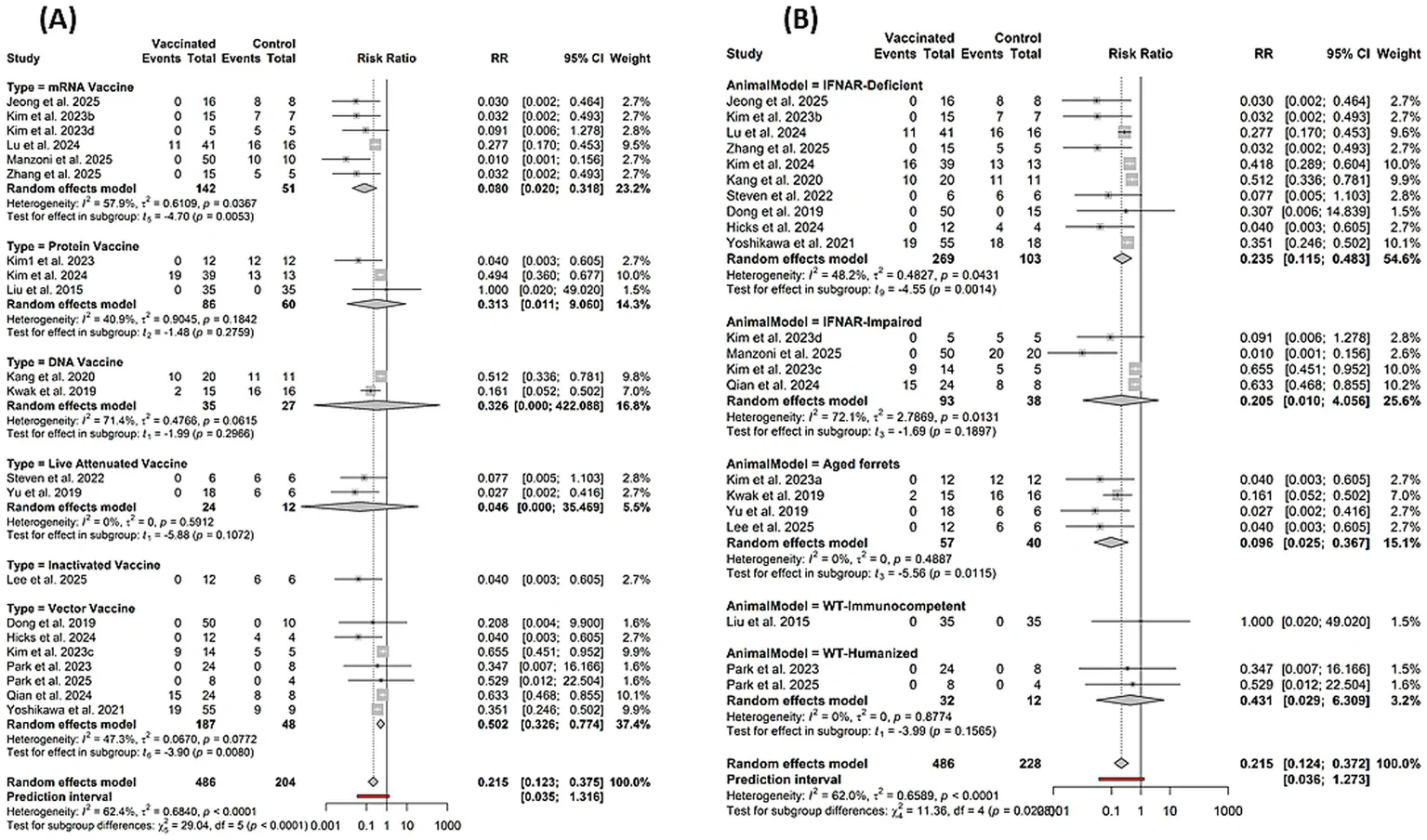
Forest Plot of subgroup analysis based on vaccine type **(A)**. Forest plot of subgroup analysis based on the animal model **(B)**. The pooled effect estimate is presented as the RR with its corresponding 95% confidence interval, calculated using a random-effects model.

Given the favorable estimates observed for mRNA and vector vaccines, further exploratory subgroup analyses were conducted to assess the influence of antigen composition, dosage frequency, and route of administration on weight-loss outcomes (Table 5). For both mRNA and vector vaccines, candidates incorporating Gn, Gc, or both glycoproteins consistently provided the greatest protection against weight loss, whereas vaccines targeting NS or NP alone showed no apparent protective effect, although this observation was based on a single study. However, subgroup differences by antigen composition were not statistically significant (*χ*^2^ = 0.18; *p* = 0.9135). Two-dose regimens were associated with a more consistent reduction in weight loss than single-dose regimens, although subgroup differences were not statistically significant (*χ*^2^ = 0.79; *p* = 0.373). Intramuscular administration showed the most consistent protective effect, whereas intradermal administration was supported by limited data, with no significant subgroup differences observed by route of administration (*χ*^2^ = 1.51; *p* = 0.219).

**Table 5:** Exploratory subgroup analysis of mRNA and Vector vaccines by vaccine antigen, dosage frequency, and vaccination route for the weight loss outcome.

Variables	Vaccine type	Subgroups	K^a^	Effect size estimates		Heterogeneity	
				RR (95% CI)	*p*-value	*I* ^2^	*p*-value
Vaccine antigen	mRNA	Gn + Gc	3	0.067 (0.001–5.345)	0.1172	73.9	0.0217
		Gn	2	0.055 (0.00–39.624)	0.1125	0	0.5928
		Gc	1	0.032 (0.002–0.493)	-	-	-
	Vector-based	Gn + Gc	5	0.247 (0.029–2.092)	0.143	64.6	0.023
		Gn	2	0.288 (0.0–582186.2)	0.4727	67	0.081
		Gc	1	1.000 (0.788–1.268)	-	-	-
		NP	1	1.000 (0.9–1.11)	-	-	-
		NSs	1	1.000 (0.022–44.831)	-	-	-
		Multi-antigen	2	0.126 (0.0–896472565.6)	0.45	55.3	0.134
Vaccine dosage	mRNA	1 dose	2	0.203 (0.0–2826560301.3)	0.545	85.8	0.008
		2 doses	6	0.039 (0.023–0.067)	<0.0001	0	0.969
	Vector-based	1 dose	4	0.435 (0.087–2.189)	0.199	25.9	0.256
		2 doses	3	0.476 (0.184–1.227)	0.0778	64.3	0.06
Vaccination route	mRNA vaccine	Intramuscular	5	0.123 (0.031–0.483)	0.0132	43.7	0.1302
		Intradermal	1	0.019 (0.001–0.315)	-	-	-
	Vector vaccine	Intravenous	1	1 (0.024–41.993)	-	-	-
		Subcutaneous	2	0.355 (0.101–1.25)	0.06	0	0.585
		Intranasal	1	1 (0.0241–41.993)	-	-	-
		Intraperitoneal	2	0.078 (0.0–12192.7)	0.224	0	0.406
		Intramuscular	4	0.64 (0.591–0.692)	0.0004	0	0.988

Among vector-based vaccines, exploratory subgroup analysis showed that antigen composition did not significantly influence weight-loss outcomes (*χ*^2^ = 5.8, *p* = 0.32). Multi-antigen and Gn + Gc-expressing vectors generally favored reduced weight loss, whereas vaccines targeting Gc or NP alone showed no apparent benefit. Dosage frequency also did not significantly affect outcomes (*χ*^2^ = 0.03, *p* = 0.873), although single-dose regimens tended to perform better than two-dose regimens. In contrast, route of administration significantly influenced protection (*χ*^2^ = 38.28, *p* < 0.0001), with intramuscular delivery associated with the greatest reduction in weight loss.

##### 3.4.3.2 Body weight trajectories

To further characterize the effects of vaccination on disease severity, longitudinal body weight trajectories were analyzed using a GLMM, accounting for repeated measurements within studies while estimating the effects of vaccine platform, challenge type, and time post-challenge. Full model estimates are presented in Supplementary Table S2, whereas model-derived estimated marginal means (EMMs) are shown in Figure 9. Across all groups, body weight declined progressively during the acute phase of infection, reaching its lowest levels approximately 6–8 days post-challenge before gradually recovering thereafter. This temporal pattern was supported by a significant overall effect of time, with the initial decline captured by the first spline term (*β* = −22.34, 95% CI: −34.50 to −10.18; *p* < 0.001). Predicted trajectories also differed according to challenge type, with homologous challenge groups consistently maintaining higher body weight than heterologous challenge groups throughout the follow-up period.

**Figure 9:**
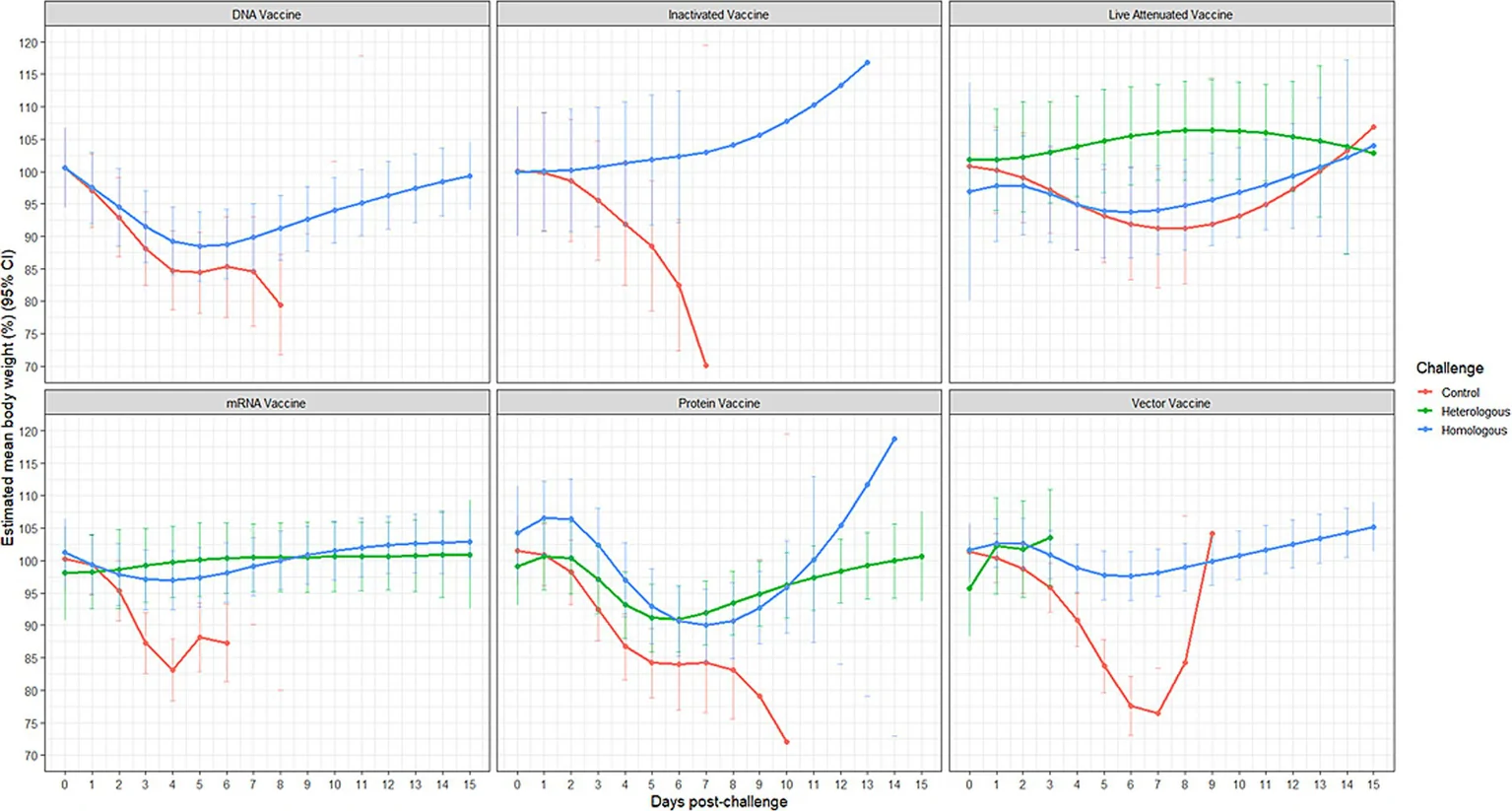
Estimated marginal means (EMMs) of longitudinal body weight change over time following SFTSV challenge by vaccine types and challenge types. EMMs of body weight (% of baseline) were derived from a generalized linear mixed model (GLMM) incorporating vaccine platform, challenge type, days post-challenge (modeled using natural splines), and their interactions, with study included as a random intercept. Each panel represents a different vaccine platform. Lines indicate the model-predicted mean body weight over time for the control (red), heterologous challenge (green), and homologous challenge (blue) groups. Error bars represent 95% confidence intervals.

Although the baseline effects of vaccine platform were not statistically significant, body weight trajectories varied over time according to vaccine platform. Significant interactions between time and mRNA vaccination were observed for the second, third, and fourth spline terms (*β* = 157.09, *p* = 0.02; *β* = −3592.17, *p* = 0.02; and *β* = −6605.82, *p* = 0.02, respectively), indicating that changes in body weight over the post-challenge period differed significantly from those of the reference platform. Similarly, vector vaccination showed a significant interaction with the second spline term (*β* = −124.44, 95% CI: −224.60 to −24.27; *p* = 0.01), supporting a distinct temporal recovery pattern. Under homologous challenge, several higher-order interactions involving mRNA vaccination remained significant, including interactions with spline terms 1–4 (*p* = 0.01–0.03), while the interaction between homologous challenge, vector vaccination, and the second spline term was also significant (*β* = 124.77, 95% CI: 24.13–225.40; *p* = 0.01). Consistent with these model estimates, the EMMs (Figure 9) demonstrated that animals vaccinated with mRNA and vector vaccines maintained their body weight closer to baseline and recovered more rapidly following challenge than those receiving the other vaccine platforms.

#### 3.4.4 Publication bias

To assess the presence of publication bias, funnel plots were constructed with the effect size on the x-axis and standard error on the y-axis. Visual inspection of the funnel plots indicated an asymmetric distribution of studies across mortality, viremia, and weight loss outcomes, suggesting the likelihood of publication bias (Figure 10). Since visual assessment of funnel plot asymmetry is inherently subjective, Egger’s regression test was performed to statistically evaluate funnel plot asymmetry. The test confirmed statistically significant small-study effects for mortality (*t* = −6.969, *p* < 0.001), viremia (*t* = −5.788, *p* < 0.001), and weight loss (*t* = −4.575, *p* < 0.001). To evaluate the potential influence of publication bias on the pooled effect size estimates, the Duval and Tweedie trim-and-fill method was used to compute adjusted effect estimates that account for potentially missing studies. For the mortality outcome, the analysis identified nine potentially missing studies. After imputing these studies, the adjusted pooled RR increased from 0.107 (95% CI: 0.054–0.213) to 0.324 (95% CI: 0.120–0.874). Despite this change in the magnitude of the effect size, the adjusted effect size remained statistically significant, indicating that publication bias did not substantially affect the overall conclusion that vaccination reduced mortality. For viremia outcome, seven potentially missing studies were imputed, and the adjusted effect size significantly shifted from RR = 0.451 (95% CI 0.276–0.737) to RR = 0.738 (95% CI: 0.361–1.509; *p* = 0.387). The loss of statistical significance after adjustment suggests that the observed protective effect of vaccination against detectable viremia may be influenced by publication bias and small-study effects. Furthermore, the trim-and-fill analysis identified nine potentially missing studies for the weight loss outcome. Imputation of these studies changed the pooled RR from 0.215 (95% CI 0.124–0.372) to 0.421 (95% CI: 0.194–0.911; *p* = 0.029). Although the magnitude of the protective effect was reduced, the adjusted estimate remained statistically significant, indicating that publication bias influenced the estimated effect size but did not alter the overall conclusion regarding the effect of vaccination on preventing weight loss.

**Figure 10:**
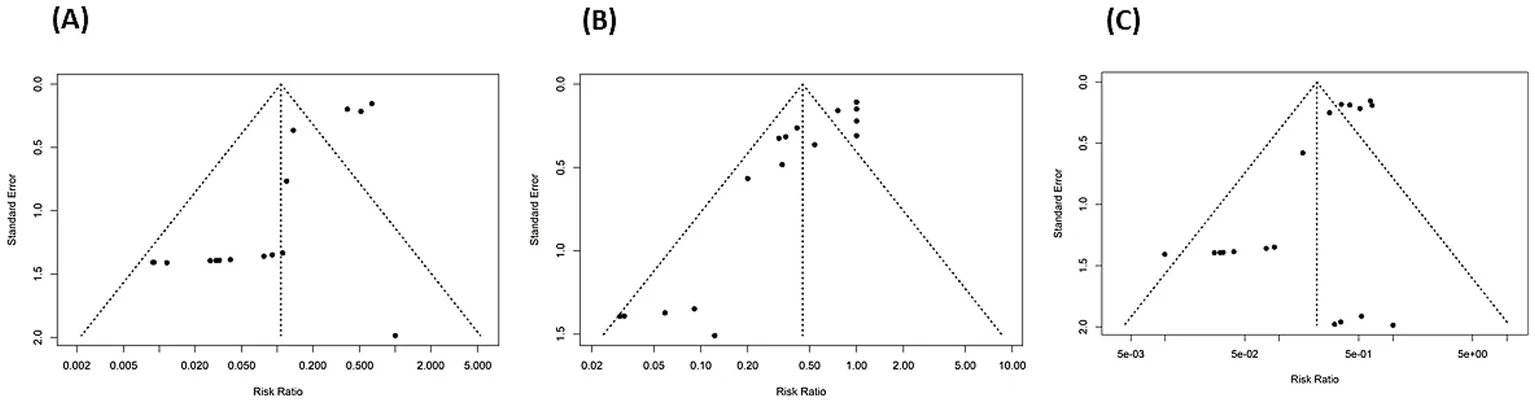
Funnel plots for publication bias on the efficacy of SFTSV vaccines in mice and ferrets considering the mortality **(A)**, viremia **(B)**, and weight loss **(C)** outcomes.

## 4 Discussion

Despite substantial progress in SFTSV vaccine development, no licensed vaccine is currently available for either humans or animals. In this study, we synthesized evidence from 23 controlled preclinical studies to provide the most comprehensive quantitative evaluation of SFTSV vaccine efficacy to date. By integrating binary outcomes with continuous measures of viral burden and longitudinal body-weight trajectories, our findings demonstrate that vaccination not only improves survival but also reduces viral replication and attenuates clinical disease following experimental SFTSV challenge.

Across the evaluated endpoints, vaccination provided the greatest and most consistent protection against mortality, with an overall vaccine efficacy of 89.3%. Mortality represents the most clinically relevant endpoint because it reflects the cumulative effects of viral replication, host immune responses, and subsequent disease progression. In SFTS, fatal outcomes are primarily associated with uncontrolled viral dissemination, excessive production of pro-inflammatory cytokines, endothelial dysfunction, and multiorgan failure rather than direct viral cytopathic effects alone (Liu et al., 2014; Park et al., 2019). The marked survival benefit observed across studies therefore suggests that vaccination can limit disease progression sufficiently to prevent fatal disease even without completely eliminating viral replication. This is consistent with previous SFTSV vaccine studies showing reduced viral replication and disease severity without necessarily inducing sterilizing immunity (Hicks et al., 2024; Kang et al., 2020). This protection is likely mediated through the coordinated induction of neutralizing antibodies against the viral Gn and Gc glycoproteins, which limit viral entry and early replication, together with virus-specific cellular immune responses that promote the elimination of infected cells and contribute to durable protective immunity (Wu et al., 2017). These findings indicate that effective vaccine-induced immunity substantially reduces the risk of fatal SFTSV infection by limiting viral replication and preventing the dysregulated host inflammatory responses that characterize severe disease.

Compared with the consistent protection against mortality, vaccination showed a more modest and heterogeneous effect on preventing detectable post-challenge viremia, with an overall vaccine efficacy of 54.9%. However, complementary analyses of viremia levels and peak viremia consistently demonstrated significant reductions in viral load following vaccination under both homologous and heterologous challenge conditions. This pattern is consistent with effective vaccine-mediated control of infection without requiring complete prevention of viral replication (Jin et al., 2012; Manzoni et al., 2025). Such protection is biologically plausible, as vaccine-induced neutralizing antibodies can rapidly restrict viral entry and dissemination, while virus-specific T-cell responses facilitate the clearance of infected cells and further limit viral amplification (Ren et al., 2024). The preservation of antiviral efficacy against heterologous challenge strains suggests that immune responses directed against relatively conserved viral antigens, particularly the Gn and Gc glycoproteins, provide meaningful cross-protection despite genetic diversity among circulating SFTSV strains. Because high viral loads are strongly associated with severe clinical disease and poor prognosis in SFTS patients (Yu et al., 2019), the observed reductions in quantitative viremia provide important mechanistic support for the substantial survival benefit observed in this meta-analysis. Collectively, these findings indicate that effective suppression of viral replication and limitation of viral burden may be important components of vaccine-mediated protection against severe and fatal SFTS, even when complete prevention of detectable infection is not achieved.

Furthermore, vaccination substantially reduced post-challenge weight loss, with an overall vaccine efficacy of 78.5%. Importantly, the longitudinal mixed-effects analysis extended this finding by demonstrating that vaccinated animals maintained higher body weight throughout the course of infection and recovered more rapidly following the acute phase than unvaccinated controls. Because body weight reflects the cumulative effects of viral replication, systemic inflammation, hematological abnormalities, and metabolic dysfunction, preservation of body weight provides an integrated measure of disease severity and clinical recovery (Yu et al., 2011; Liu et al., 2014). Together with the marked reductions in viral burden observed in this meta-analysis, these findings suggest that vaccination not only limits viral replication but also mitigates the downstream immunopathological processes responsible for clinical illness.

Subgroup analyses provided exploratory evidence of variation in vaccine efficacy according to platform, animal model, antigen composition, vaccine regimen, and administration route. Favorable efficacy estimates were frequently observed for mRNA- and vector-based vaccines, particularly those expressing the SFTSV Gn and Gc glycoproteins. This likely reflects their ability to induce coordinated humoral and cellular immune responses through efficient antigen expression and presentation, while targeting the major viral surface glycoproteins responsible for host-cell attachment and membrane fusion (Park et al., 2023; Zhang et al., 2025). In contrast, protein subunit and inactivated vaccines primarily rely on exogenous antigen presentation and may elicit comparatively weaker cellular immunity (Kim et al., 2024). However, given the limited number of studies contributing to several subgroups, these findings should be considered hypothesis-generating rather than confirmatory evidence of superiority of any particular vaccine platform or antigen. Strong protection observed in IFNAR-deficient mice and aged ferrets demonstrates the robustness of vaccine-induced protection in highly susceptible models of severe SFTSV infection, but findings from IFNAR-deficient mice may not directly predict protection in immunocompetent older adults. Further studies in immunocompetent, aged, and humanized models are needed to strengthen translational relevance. Moreover, the exploratory observation that two-dose vaccination regimens and intramuscular administration were associated with greater protection across several endpoints is consistent with established immunological principles, whereby prime–boost immunization enhances immune memory and affinity maturation, while intramuscular delivery promotes efficient antigen uptake and activation of adaptive immune responses. Although these findings require confirmation in standardized comparative studies, they provide valuable guidance for the rational design and optimization of next-generation SFTSV vaccines.

Overall, vaccination was associated with significant protective effects against mortality, detectable viremia, and post-challenge weight loss following SFTSV infection. However, these findings should be interpreted cautiously, particularly when extrapolating beyond the experimental settings represented in the included studies. The substantial between-study heterogeneity, together with the relatively wide confidence and prediction intervals observed across outcomes, indicates considerable uncertainty in the magnitude of the estimated vaccine effects and suggests that effects may vary under different experimental conditions. Accordingly, the pooled estimates should be interpreted as average effects across the available preclinical evidence, rather than as universally applicable effect sizes. While the consistent direction of the observed effects supports an overall protective effect of vaccination, careful extrapolation to other animal models, experimental conditions, or clinical settings is warranted.

Taken together, these findings have important implications for both vaccine development and broader One Health strategies for SFTS control. Beyond individual protection, reductions in systemic viral burden may also influence SFTSV transmission within tick–animal cycles involving wildlife, livestock, and companion animals (Chen et al., 2019). However, the viremia level and duration required for efficient tick acquisition and onward transmission remain unknown, limiting assessment of potential vaccine-mediated transmission-blocking effects. Vaccinated dogs have shown strong neutralizing responses and no detectable viremia following challenge, suggesting reduced availability of virus for tick acquisition (Park et al., 2024). In goats, experimental and natural infection studies similarly resulted in transient viremia lasting less than 24 h, with no detectable viral RNA in respiratory or digestive secretions; however, viral RNA was detected in feeding ticks during periods associated with goat viremia, and identical SFTSV S-segment sequences were recovered from an infected goat and its feeding *Haemaphysalis longicornis* tick (Jiao et al., 2015). These findings support the potential relevance of host viremia to tick acquisition but do not establish a quantitative viremia threshold for transmission. Dedicated studies measuring tick acquisition and onward transmission across defined levels and durations of viremia are therefore needed to determine whether vaccination can disrupt the animal–tick–animal transmission cycle and contribute to integrated One Health approaches for reducing SFTSV circulation in endemic regions.

This study has a few limitations that should be acknowledged. Although the search strategy was comprehensive and imposed no language restrictions, the exclusive use of English search terms may have resulted in the omission of relevant studies published in other languages. We also acknowledge that we did not plan to contact vaccine companies or research institutes for unpublished data that could have been eligible for inclusion. It is also important to note that the meta-analysis indicated potential publication bias for detectable viremia. Vaccination was associated with reduced detectable viremia (RR = 0.45, 95% CI 0.27–0.73, *p* = 0.003), although this effect became non-significant after trim-and-fill adjustment (adjusted RR = 0.74, 95% CI 0.36–1.51, *p* = 0.387). This shift suggests that the observed protective effect may have been influenced by small-study effects and potentially missing studies, consistent with the well-recognized “file drawer” problem, whereby studies with non-significant or unfavorable findings may be less likely to be published (Lishner, 2022). However, the presence of publication bias should be interpreted cautiously, as funnel-plot asymmetry may also arise from genuine between-study heterogeneity and other differences among preclinical studies rather than true selective reporting. Accordingly, the trim-and-fill estimate should be interpreted as a sensitivity analysis rather than a definitive correction for publication bias (Peters et al., 2007). In addition, no study was judged to be at low risk of bias across all assessed domains, mainly because of insufficient reporting of sample size determination and blinding procedures. Although these domains were classified as unclear rather than high risk, this incomplete reporting reduces confidence in the pooled estimates and warrants cautious interpretation. Furthermore, while this review expanded beyond binary endpoints by incorporating quantitative analyses of viremia levels, peak viremia, and longitudinal body-weight trajectories, these analyses were constrained by inconsistent reporting of these outcomes across primary studies, thereby reducing the number of studies available for inclusion. Future studies should therefore adopt standardized reporting of virologic and clinical outcomes in experimental animals, incorporate tissue-specific viral load, durability assessments, and evaluate cross-protection against genetically diverse SFTSV strains. Addressing these gaps will be important for translating the promising protection observed in experimental models into effective vaccines for relevant human and veterinary populations.

## 5 Conclusion

This systematic review and meta-analysis provide comprehensive quantitative evidence that vaccination can effectively protect against severe SFTSV infection in animal models. Across complementary clinical and virological outcomes, vaccination markedly improved survival, suppressed viral replication, and mitigated disease severity, supporting control of viral replication rather than complete sterilizing immunity as a key determinant of protection. Among the evaluated strategies, mRNA and vector-based vaccines, particularly those targeting the Gn and Gc glycoproteins, demonstrated the most favorable efficacy profiles, providing quantitative evidence to inform future platform and antigen selection beyond the largely study-specific and narrative evidence available to date. However, the current evidence remains predominantly preclinical, with limited evaluation in veterinary target species and important gaps regarding durability of protection, immune correlates, and cross-strain efficacy. Addressing these gaps through standardized and translationally relevant studies, including evaluation in immunocompetent and humanized models and against heterologous strains, will be essential to advance the most promising candidates toward broadly protective SFTSV vaccines for both human and veterinary use within a One Health framework.

## Data Availability

The original contributions presented in the study are included in the article/Supplementary material, further inquiries can be directed to the corresponding author/s.
