# Supplementary material for: Efficacy of severe fever with thrombocytopenia syndrome vaccines in animal models: a systematic review and meta-analysis
Source: Front Microbiol. 2026 Oct 13;17:1978600. doi: 10.3389/fmicb.2026.1978600 (PMC13710250; doi:10.3389/fmicb.2026.1978600)
Supplement: Supplementary file 1 [file Table_2.DOCX]

**Table S2: Summary of the characteristics of the vaccine protocols and challenges experiments included in the analysis**

| **Author** | **Vaccine Type** | **Vaccine antigen** | **Adjuvant** | **Dose** | **Dose Frequency** | **Timing of vaccination** | **Challenge viral strain** | **Viral Challenge titer** | **Time between vaccination & challenge** | **Challenge administration route** | **Follow-up** |
| --- | --- | --- | --- | --- | --- | --- | --- | --- | --- | --- | --- |
| Jeong et al. 2025 | mRNA Vaccine | Gn+Gc | None | 2μg | 2-dose | 0-3 weeks | 2015-JJ01 | 1 × 10^3^ PFU | 2 weeks | Subcutaneous | 14 days |
|  | mRNA Vaccine | Gn+Gc | None | 20μg | 2-dose | 0-3 weeks | 2015-JJ01 | 1 × 10^3^ PFU | 2 weeks | Subcutaneous | 14 days |
| Kim et al. 2023b | mRNA Vaccine | Gn | None | 3μg | 2-dose | 0-3 weeks | HB29 | 2 × 10⁴ PFU | 2 weeks | Intramuscular | 15 days |
|  | mRNA Vaccine | Gn | None | 3μg | 2-dose | 0-3 weeks | HB29 | 2 × 10⁴ PFU | 2 weeks | Intramuscular | 15 days |
| Kim et al. 2023d | mRNA Vaccine | Gn | None | 10μg | 2-dose | 0-14 days | Korean Isolate (MN329148–150) | 1 × 10^5^ FFU | 3 weeks | Subcutaneous | 14 days |
| Lu et al. 2024 | mRNA Vaccine | Gn+Gc | None | 1µg | 1-dose | Day 0 | A129 | 1 × 10^5^ TCID_50_ | 10 days | Intraperitoneal | 14 days |
|  | mRNA Vaccine | Gn+Gc | None | 1µg | 2-dose | 0-3 weeks | A129 | 1 × 10^5^ TCID_50_ | 10 days | Intraperitoneal | 14 days |
|  | mRNA Vaccine | Gn+Gc | None | 0.1µg | 2-dose | 0-3 weeks | A129 | 1 × 10^5^ TCID_50_ | 10 days | Intraperitoneal | 14 days |
|  | mRNA Vaccine | Gn+Gc | None | 1µg | 2-dose | 0-3 weeks | A129 | 1 × 10^5^ TCID_50_ | 10 days | Intraperitoneal | 14 days |
|  | mRNA Vaccine | Gn+Gc | None | 5µg | 2-dose | 0-3 weeks | A129 | 1 × 10^5^ TCID_50_ | 10 days | Intraperitoneal | 14 days |
| Manzoni et al. 2025 | mRNA Vaccine | Gn+Gc | None | NR | 1-dose | Day -14 | HB29 | 26,000 CCID₅₀ | 14 days | Intraperitoneal | 14 days |
|  | mRNA Vaccine | Gn+Gc | None | NR | 1-dose | Day -14 | HB29 | 26,000 CCID₅₀ | 14 days | Intraperitoneal | 14 days |
|  | mRNA Vaccine | Gn+Gc | None | NR | 2-dose | -35-14 days | HB29 | 26,000 CCID₅₀ | 14 days | Intraperitoneal | 14 days |
|  | mRNA Vaccine | Gn+Gc | None | NR | 2-dose | -35-14 days | HB29 | 26,000 CCID₅₀ | 14 days | Intraperitoneal | 14 days |
|  | mRNA Vaccine | Gn+Gc | None | NR | 2-dose | -35-14 days | HB29 | 26,000 CCID₅₀ | 14 days | Intraperitoneal | 14 days |
| Zhang et al. 2025 | mRNA Vaccine | Gc | None | 10μg | 2-dose | 0-3 weeks | HBMC5 | 1 * 10^3^ FFU | 3 weeks | Subcutaneous | 21 days |
|  | mRNA Vaccine | Gc | None | 5μg | 2-dose | 0-3 weeks | HBMC5 | 1 * 10^3^ FFU | 3 weeks | Subcutaneous | 21 days |
|  | mRNA Vaccine | Gc | None | 1μg | 2-dose | 0-3 weeks | HBMC5 | 1 * 10^3^ FFU | 3 weeks | Subcutaneous | 21 days |
| Kim et al. 2023a | Protein Vaccine | Gn | AddaVax | 15µg | 3-dose | 0-3-6 weeks | DBV CB1/2014 | 10^7.6^ TCID_50_/ml | 2 weeks | Intramuscular | 14 days |
| Kim et al. 2024 | Protein Vaccine | NP | Aluminum hydroxy chloride | 10µg | 3-dose | 0-14-28 days | 2015-JJ01 | 10^3^ FFU | 2 weeks | Subcutaneous | 14 days |
|  | Protein Vaccine | Gn | Aluminum hydroxy chloride | 10µg | 3-dose | 0-14-28 days | 2015-JJ01 | 10^3^ FFU | 2 weeks | Subcutaneous | 14 days |
|  | Protein Vaccine | Gc | Aluminum hydroxy chloride | 10µg | 3-dose | 0-14-28 days | 2015-JJ01 | 10^3^ FFU | 2 weeks | Subcutaneous | 14 days |
|  | Protein Vaccine | NP+Gn | Aluminum hydroxy chloride | 10µg | 3-dose | 0-14-28 days | 2015-JJ01 | 10^3^ FFU | 2 weeks | Subcutaneous | 14 days |
|  | Protein Vaccine | NP+Gc | Aluminum hydroxy chloride | 10µg | 3-dose | 0-14-28 days | 2015-JJ01 | 10^3^ FFU | 2 weeks | Subcutaneous | 14 days |
|  | Protein Vaccine | Multi-antigen | Aluminum hydroxy chloride | 10µg | 3-dose | 0-14-28 days | 2015-JJ01 | 10^3^ FFU | 2 weeks | Subcutaneous | 14 days |
| Liu et al. 2015 | Protein Vaccine | NSs | Freund’s adjuvant | 100µg prime+ 50µg boost | 2-dose | 1-14 days | WCH/97/HN/China/2011 | 3 · 10^7^ FFU | 26 days | Intraperitoneal | 28 days |
| Kang et al. 2020 | DNA Vaccine | Multi-antigen | None | 4µg | 3-dose | 1-14-28 days | Korean SFTSV isolate | 10^5^ FFU | 2 weeks | Subcutaneous | 10 days |
|  | DNA Vaccine | Multi-antigen | None | 4µg | 3-dose | 1-14-28 days | Korean SFTSV isolate | 10^5^ FFU | 2 weeks | Subcutaneous | 10 days |
|  | Protein Vaccine | Gn | Alum (Alhydrogel 2%) | 20µg | 3-dose | 1-14-28 days | Korean SFTSV isolate | 10^5^ FFU | 2 weeks | Subcutaneous | 10 days |
|  | Protein Vaccine | Gc | Alum (Alhydrogel 2%) | 20µg | 3-dose | 1-14-28 days | Korean SFTSV isolate | 10^5^ FFU | 2 weeks | Subcutaneous | 10 days |
| Kwak et al. 2019 | DNA Vaccine | Multi-antigen | None | 200µg | 3-dose | -42-28-14 days | CB1/2014 | 10^7.6^ TCID_50_ | 2 weeks | Intramuscular | 14 days |
|  | DNA Vaccine | Gn+Gc | None | 200µg | 3-dose | -42-28-14 days | CB1/2014 | 10^7.6^ TCID_50_ | 2 weeks | Intramuscular | 14 days |
|  | DNA Vaccine | Multi-antigen | None | 200µg | 3-dose | -42-28-14 days | CB1/2014 | 10^7.6^ TCID_50_ | 2 weeks | Intramuscular | 14 days |
| Steven et al. 2022 | Live Attenuated Vaccine | Whole virus | None | 10^5^ FFU | 1-dose | Day 0 | wt HB29 | 10^5^ FFU | 14 days | Subcutaneous | 14 days |
| Yu et al. 2019 | Live Attenuated Vaccine | Whole virus | None | 4 × 10^6^ PFUs | 1-dose | 1 day | CB1/2014 | 5 × 10^7^ PFU | 8 weeks | Intramuscular | 10 days |
|  | Live Attenuated Vaccine | Whole virus | None | 4 × 10^6^ PFUs | 1-dose | 1 day | CB1/2014 | 5 × 10^7^ PFU | 8 weeks | Intramuscular | 10 days |
|  | Live Attenuated Vaccine | Whole virus | None | 5 × 10^5^ PFUs | 1-dose | 1 day | CB1/2014 | 5 × 10^7^PFU | 8 weeks | Intramuscular | 10 days |
| Li et al. 2022 | Inactivated Vaccine | Whole virus | Al(OH)₃ | 4µg | 3-dose | 0-14-28 days | HB29 | 10^5^ TCID_50_ | 14 days | Intramuscular | 14 days |
|  | Inactivated Vaccine | Whole virus | Al(OH)₃ | 1µg | 3-dose | 0-14-28 days | HB29 | 10^5^ TCID_50_ | 14 days | Intramuscular | 14 days |
|  | Inactivated Vaccine | Whole virus | Al(OH)₃ | 0.25µg | 3-dose | 0-14-28 days | HB29 | 10^5^ TCID_50_ | 14 days | Intramuscular | 14 days |
| Lee et al. 2025 | Inactivated Vaccine | Whole virus | None | 30µg | 2-dose | 0-14 days | CB1/2014 | 10^6.0^ TCID_50_ mL−1 | 30 weeks | Intramuscular | 14 days |
|  | Inactivated Vaccine | Whole virus | Alum | 30µg | 2-dose | 0-14 days | CB1/2014 | 10^6.0^ TCID_50_ mL−2 | 30 weeks | Intramuscular | 14 days |
| Dong et al. 2019 | Vector Vaccine | Gn+Gc | None | (30ul) 1x104 PFU | 1-dose | Day 0 | Wuhan | 2 × 10^4^ FFU | 30 days | Intraperitoneal | 14 days |
|  | Vector Vaccine | Gn+Gc | None | (100ul) 1x104 PFU | 1-dose | Day 0 | Wuhan | 2 × 10^4^ FFU | 30 days | Intraperitoneal | 14 days |
|  | Vector Vaccine | Gn+Gc | None | (30ul) 1x104 PFU | 1-dose | Day 0 | Wuhan | 2 × 10^4^ FFU | 30 days | Intraperitoneal | 14 days |
|  | Vector Vaccine | Gn+Gc | None | (100ul) 1x104 PFU | 1-dose | Day 0 | Wuhan | 2 × 10^4^ FFU | 30 days | Intraperitoneal | 14 days |
|  | Vector Vaccine | Gn+Gc | None | 2 × 10^1^ PFU | 1-dose | Day 0 | Wuhan | 2 × 10^4^ FFU | 30 days | Intraperitoneal | 14 days |
|  | Vector Vaccine | Gn+Gc | None | 2 × 10^2^ PFU | 1-dose | Day 0 | Wuhan | 2 × 10^4^ FFU | 30 days | Intraperitoneal | 14 days |
|  | Vector Vaccine | Gn+Gc | None | 2 × 10^3^ PFU | 1-dose | Day 0 | Wuhan | 2 × 10^4^ FFU | 30 days | Intraperitoneal | 14 days |
|  | Vector Vaccine | Gn+Gc | None | 2 × 10^4^ PFU | 1-dose | Day 0 | Wuhan | 2 × 10^4^ | 30 days | Intraperitoneal | 14 days |
|  | Vector Vaccine | Gn+Gc | None | 2 × 10^4^ PFU | 1-dose | Day 0 | Wuhan | 2 × 10^4^ FFU | 30 days | Intraperitoneal | 14 days |
|  | Vector Vaccine | Gn+Gc | None | 2 × 10^4^ PFU | 1-dose | Day 0 | Wuhan | 2 × 10^4^ FFU | 30 days | Intraperitoneal | 14 days |
| Hicks et al. 2024 | Vector Vaccine | Gn+Gc | None | 10^4^ PFU | 1-dose | Day 0 | HB29 | 10 PFU | 23 days | Subcutaneous | 21 days |
|  | Vector Vaccine | Gn+Gc | None | 10^3^ PFU | 1-dose | Day 0 | HB29 | 10 PFU | 23 days | Subcutaneous | 21 days |
|  | Vector Vaccine | Gn+Gc | None | 10^2^ PFU | 1-dose | Day 0 | HB29 | 10 PFU | 23 days | Subcutaneous | 21 days |
| Kim3 et al. 2023 | Vector Vaccine | Gn | Alum | 5μg | 2-dose | 0-14 days | MN329148–MN329150 | 1 × 10^5^ FFU | 5 weeks | Subcutaneous | 6 days |
|  | Vector Vaccine | Gn | Alum | 1 × 10^8^ PFU | 2-dose | 0-14 days | MN329148–MN329150 | 1 × 10^5^ FFU | 5 weeks | Subcutaneous | 6 days |
|  | Vector Vaccine | Gn | Alum | 1 × 10^8^ PFU | 2-dose | 0-14 days | MN329148–MN329150 | 1 × 10^5^ FFU | 5 weeks | Subcutaneous | 6 days |
| Park et al. 2023 | Vector Vaccine | Multi-antigen | None | 1 × 10⁷ CFU / 100 Μl | 2-dose | 0-14 days | KADGH (NCCP43261) | 1 × 10^3^ FAID_50_ | 2 weeks | Intraperitoneal | 4 days |
|  | Vector Vaccine | Multi-antigen | None | 1 × 10⁷ CFU / 100 Μl | 2-dose | 0-14 days | KADGH (NCCP43261) | 1 × 10^3^ FAID_50_ | 2 weeks | Intraperitoneal | 4 days |
|  | Vector Vaccine | NSs | None | 1 × 10⁷ CFU / 100 Μl | 2-dose | 0-14 days | KADGH (NCCP43261) | 1 × 10^3^ FAID_50_ | 2 weeks | Intraperitoneal | 4 days |
| Park et al. 2025 | Vector Vaccine | Gn+Gc | None | 1 × 10^7^ CFU | 1-dose | Day 0 | SFTSV strain | 1 × 10^4^ FAID_50_ | 2 weeks | Intraperitoneal | 4 days |
|  | Vector Vaccine | Gn+Gc | None | 1 × 10^7^ CFU | 1-dose | Day 0 | SFTSV strain | 1 × 10^4^ FAID_50_ | 2 weeks | Intraperitoneal | 4 days |
| Qian et al. 2024 | Vector Vaccine | Gn | None | 10^8^ PFU/ml | 1-dose | Day 0 | JS2011-013-1 | 1 × 10^5.25^TCID_50_ | 28 days | Intraperitoneal | 21 days |
|  | Vector Vaccine | Gc | None | 10^8^ PFU/ml | 1-dose | Day 0 | JS2011-013-1 | 1 × 10^5.25^TCID_50_ | 28 days | Intraperitoneal | 21 days |
|  | Vector Vaccine | Gn+Gc | None | 10^8^ PFU/ml | 1-dose | Day 0 | JS2011-013-1 | 1 × 10^5.25^TCID_50_ | 28 days | Intraperitoneal | 21 days |
| Tian et al. 2021 | Vector Vaccine | Gn | None | 10^7^ FFU | 1-dose | Day 0 | JS2011-013-1 | 2 × 10^7^ TCID_50_ | 3 weeks | Intramuscular | 7 days |
| Yoshikawa et al. 2021 | Vector Vaccine | NP | None | 1×10⁶ PFU | 2-dose | 0-14 days | YG-1 | 1 × 10^3^ TCID_50_ | 2 weeks | Subcutaneous | 14 days |
|  | Vector Vaccine | Gn+Gc | None | 1×10⁶ PFU | 2-dose | 0-14 days | YG-1 | 1 × 10^3^ TCID_50_ | 2 weeks | Subcutaneous | 14 days |
|  | Vector Vaccine | Multi-antigen | None | 1×10⁶ PFU | 2-dose | 0-14 days | YG-1 | 1 × 10^3^ TCID_50_ | 2 weeks | Subcutaneous | 14 days |
|  | Vector Vaccine | NP | None | 1×10⁶ PFU | 2-dose | 0-14 days | YG-1 | 1 × 10^5^ TCID_50_ | 2 weeks | Subcutaneous | 14 days |
|  | Vector Vaccine | Gn+Gc | None | 1×10⁶ PFU | 2-dose | 0-14 days | YG-1 | 1 × 10^5^ TCID_50_ | 2 weeks | Subcutaneous | 14 days |
|  | Vector Vaccine | Multi-antigen | None | 1×10⁶ PFU | 2-dose | 0-14 days | YG-1 | 1 × 10^5^ TCID_50_ | 2 weeks | Subcutaneous | 14 days |
